# THY1 (CD90) Maintains the Adherens Junctions in Nasopharyngeal Carcinoma via Inhibition of SRC Activation

**DOI:** 10.3390/cancers15072189

**Published:** 2023-04-06

**Authors:** Luo Chen, Wai Yin Chau, Hei Tung Yuen, Xiao Han Liu, Robert Zhong Qi, Maria Li Lung, Hong Lok Lung

**Affiliations:** 1 Department of Chemistry, Faculty of Science, Hong Kong Baptist University, Hong Kong 999077, China; 2 Bioscience and Biomedical Engineering Thrust, The Hong Kong University of Science and Technology (Guangzhou), Guangzhou 511400, China; 3 Division of Life Science, The Hong Kong University of Science and Technology, Hong Kong 999077, China; 4 Department of Clinical Oncology, University of Hong Kong, Hong Kong 999077, China

**Keywords:** THY1, CD90, SRC, NPC, PDGF-Rβ, PTPN22, adherens junctions

## Abstract

**Simple Summary:**

Nasopharyngeal carcinoma (NPC) is endemic to southern China and cancer metastasis remains the main cause of treatment failures. Previously we have found THY1, a cell surface glycoprotein, inhibits the invasion of NPC cells and functions as a tumor suppressor in NPC. In the present study, we further illustrated the mechanism that contributes to the tumor suppressive function of THY1. Two binding partners of THY1, PDGF-Rβ and PTPN22, were identified, and PTPN22 represents the downstream signaling molecule of THY1 to inhibit the PDGF-Rβ–induced SRC activity. The anti-metastatic effect of SRC inhibition was subsequently validated in a mice model with administration of a SRC inhibitor that has been used in clinics for other diseases. This study opens a new window to target the SRC signaling activity which is antagonized by THY1 in NPC.

**Abstract:**

We had previously shown that THY1 (CD90) is a tumor suppressor in nasopharyngeal carcinoma (NPC) and that its down-regulation and loss of expression are associated with tumor metastasis, yet the mechanism leading to such effects remains unknown. In this study we show that tumor invasion could be suppressed by THY1 via adherens junction formation in a few NPC cell lines, and knockdown of THY1 would disrupt this cell-cell adhesion phenotype. Mechanistically, the activity of the SRC family kinase (SFK) member, SRC, and canonical Wnt signaling were dramatically reduced when THY1 was constitutively expressed. Previous studies by others have found that high levels of SRC activity in NPCs are associated with EMT and a poor prognosis. We hypothesized that THY1 can suppress tumor invasion in NPC via inhibition of SRC. By gene silencing of SRC, we found that the in vitro NPC cell invasion was significantly reduced and adherens junctions were restored. Through proteomic analysis, we identified that platelet-derived growth factor receptor β (PDGF-Rβ) and protein tyrosine phosphatase nonreceptor type 22 (PTPN22) are novel and potential binding partners of THY1, which were subsequently verified by co-immunoprecipitation (co-IP) analysis. The ligand of PDGF-Rβ (PDGF-BB) could highly induce SRC activation and NPC cell invasion, which could be almost completely suppressed by THY1 expression. On the other hand, the PTPN22 siRNA could enhance both the SRC activities and the cell invasion and could also disrupt the adherens junctions in the THY1-expressing NPC cells; the original THY1-induced phenotypes were reverted when the PTPN22 expression was reduced. Together, our results identified that PTPN22 is essential for THY1 to suppress cell invasion and SRC activity, maintain tight adherens junctions, and prevent NPC metastasis. These results suggested that PDGF-Rβ and SRC can be used as drug targets for suppressing NPC metastasis. Indeed, our in vivo assay using the SRC inhibitor KX2-391, clearly showed that inhibition of SRC signaling can prevent the metastasis of NPC, indicating that targeting SRC can be a promising approach to control the NPC progression.

## 1. Introduction

Nasopharyngeal carcinoma (NPC), which is derived from the epithelium of the nasopharynx, is a malignant tumor that invades nearby anatomical spaces or organs [1]. The distribution of NPC is regionally biased, which causes a huge burden in endemic regions. In 2018, there were 129,000 new NPC cases globally, and more than 70% of those cases came from Southeast and East Asia [2]. Conventional radiotherapy and chemotherapy are still the major treatment modalities for NPC; however, NPC patients commonly present advanced disease at diagnosis, and treatments become relatively ineffective. A key challenge in the clinical management of NPC is the development of distant failure and the lack of effective therapy. Metastatic NPC is extremely difficult to manage, with a mortality rate of over 91% within the 1st year of metastasis [3]. Therefore, preventing tumor metastasis can improve the survival of NPC patients. Our previous studies have identified that THY1 is a tumor suppressor downregulated in NPC via promoter hypermethylation, and such downregulated THY1 expression has been linked to more severe NPC lymph node metastasis [4,5]. From these studies, it was found that THY1 can inhibit in vitro cell invasion, but the underlying mechanism still remains unclear. In this study, such mechanism was investigated and whether the THY1 downstream signaling pathway could be used as a drug target was examined.

THY1 (or CD90) is a 25–37 kDa cell surface glycoprotein that is anchored to the plasma membrane by the glycosylphosphatidylinositol (GPI) motif at the C-terminus; it lacks both the transmembrane and cytoplasmic domains and well-defined ligands [6]. It was originally recognized as a thymocyte marker, since THY1 was found to be ambiguously expressed in a wide range of cell types with diverse biological functions including tumor suppression, regulation of adipogenesis, cell-cell and cell-matrix adhesion, cell migration, cell differentiation, and T-cell activation [6]. However, its physiological roles in mammalians are poorly understood, as the THY1-knockout mouse phenotype was not clearly defined [7]. THY1 can induce some biological functions, such as the control of adipogenesis through the regulation of SRC family kinases (SFKs) [8,9]. In fibroblasts, THY1 was shown to inhibit SFK activation and result in increased focal adhesions and reduced wound healing capability [10]. Yet to our knowledge, no study has been conducted to investigate the interaction between THY1 and SRC in NPC cancer development or the functional relevance of their interaction. In this study, we examined the effects of THY1 on SRC activity with both forced expression of THY1 and shRNA-mediated knockdown of THY1 in NPC cell models.

SRC (or C-SRC) is a non-receptor tyrosine kinase and is recognized as a proto-oncogene belonging to SFKs, which include SRC, LYN, FYN, YES, BLK, YRK, FGR, HCK, and LCK. The general structure of SFKs includes a N-terminus (SH4 domain) with lipid modifications, which targets the SFKs to the cytoplasmic membrane for interaction with transmembrane receptor tyrosine kinases (RTKs) [11] such as platelet-derived growth factor receptor (PDGF-R) [12], followed by the regulatory SH3 and SH2 domains, a catalytic protein tyrosine kinase (PTK) core, and a C-terminus regulatory tail that contains the hallmark tyrosine residue (Y530 in human SRC) [13,14]. The regulation of kinase activity is controlled by multiple intermolecular interactions and by the phosphorylation and dephosphorylation of two critical tyrosine residues: the activating tyrosine residue (Y419 in human SRC, Y416 in mouse SRC) in the PTK domain, and the regulatory/inhibitory residue (Y530 in human SRC, Y527 in mouse SRC) at the C-terminus [14,15]. Phosphorylation at SRC^Y419^ in the PTK domain up-regulates the SRC enzyme activity, while phosphorylation at SRC^Y530^ in the C-terminal tail by C-SRC tyrosine kinase (CSK) reduces the enzymatic activities.

The elevated activities of SRC are associated with the development and progression of a number of cancers by promoting oncogenic signals. The activation of SRC can disrupt epithelial cell-cell junctions and induce epithelial-mesenchymal transition (EMT), which contributes to tumor cell invasion and cancer progression [16]. In NPC, elevated levels of SRC in serum and activated form of SRC in primary tissues were found to be associated with poor prognosis; the gain-of-function and loss-of-function analyses have demonstrated that SRC could induce EMT and enhance the metastasis potential in the EBV-negative CNE2 and SUNE1 cell lines used as the study models [17]. This is one of the very few evidence findings to reveal the functional role(s) of SRC in facilitating the malignancy of NPC. In this study, we first demonstrated that the NPC tumor suppressor THY1 could inhibit the activation of SRC. Such inhibition was accompanied by inhibited Wnt signaling, restoration of adherens junctions, and suppressed tumor invasion. Using proteomic profiling of the THY1 binding partners, platelet-derived growth factor receptor β (PDGF-Rβ), an activator of SFKs, and protein tyrosine phosphatase nonreceptor type 22 (PTPN22), an inhibitor of SFKs, were found to be candidate binding proteins of THY1. The THY1/PDGF-Rβ and THY1/PTPN22 interactions were subsequently validated by the co-IP analyses in a NPC cell line and an immortalized epithelial cell line, supporting that THY1 can regulate SRC activity in NPC via interaction with those two SRC modulators. Lastly, a panel of SRC inhibitors were screened for growth inhibition on NPC cells, and the best candidate, KX2-391, was chosen for further NPC lung metastasis assay and was found to inhibit NPC metastasis. Taken together, this paper sought to investigate the underlying mechanisms of THY1-mediated anti-metastatic effects in NPC, and our data showed that THY1 could inhibit the SRC activity via interaction with PDGF-Rβ and PTPN22 in NPC cells, so as to protect the adherens junctions and suppress NPC metastasis.

## 2. Materials and Methods

### 2.1. Cell Culture and Chemicals

HONE1, HK1, CNE1, CNE2, SUNE1, C666-1, and NPC43 NPC cell lines, and immortalized nasopharyngeal epithelial cell lines NP69 and NP460 were cultured as previously described [4,18]. Cell lines were authenticated by and obtained from the Hong Kong NPC AoE Cell Line Repository. HK11.19 is a HONE1/chromosome 11 microcell hybrid cell line (MCH) that was established by the transfer of an additional intact human chromosome 11 into HONE1 and expressed physiological levels of THY1 (in the recipient HONE1 cells, THY1 expression is downregulated by promoter hymermethylation, and human THY1 maps to chromosome 11q22.3) [4,19]. Recombinant human PDGF-BB was obtained from PeproTech (PeproTech, Rocky Hill, NJ, USA, #100-14B-10). KX2-391 (Selleckchem, Houston, TX, USA, #S2700), bosutinib (Selleckchem, Houston, TX, USA, #S1014), and saracatinib (Selleckchem, Houston, TX, USA, #1006) were prepared in DMSO at a concentration of 10 mM for an in vitro assay. For the in vivo assay, KX2-391 was first dissolved in DMSO to a concentration of 85 mg/mL, then formulated with PEG-300 (Sigma-Aldrich, St. Louis, MI, USA, #202371) and PBS before injection following the manufacturer’s instructions (the injection solution contains 4% DMSO, 30% PEG-300, and 66% PBS).

### 2.2. Establishment of THY1-Expressing NPC Cell Lines

HONE1, HK1, and NPC43 cells with low THY1 expression were used as recipient cells for THY1 restoration with stable expression of THY1, mediated by the pLVX expression vector [20]. The packaging vectors psPAX2 and pMD2.G were obtained from Addgene. The pLVX-EF1α-THY1-expressing vector was constructed. Lentivirus packaging was performed using 293T cells and X-tremeGENE transfection reagent (Millipore, Burlington, MA, USA, #6366236001). The cells were incubated for the production of lentivirus-containing medium for 2 days. Subsequently, the medium was harvested and stored at −80 °C until usage. For infection, NPC cells were trypsinized and then mixed with the lentivirus-containing medium at a ratio of 1:1 in a culture flask, with the addition of polybrene (Santa Cruz, Santa Cruz, CA, USA, #sc-134220). After 12 h, the medium was replaced with fresh, complete medium. Puromycin (Invitogen, Waltham, MA, USA, #ant-pr-1) was used for selection of successfully infected cells.

### 2.3. Microscopy for Immunofluorescence (IF) Stained Samples and for Living Cells

IF staining was performed as previously described [18]. Briefly, cells were grown on coverslips, fixed with 10% formalin at room temperature for 15 min, washed with PBS three times, permeabilized with 0.2% Triton X-100 for 15 min, blocked with 3% bovine serum albumin (BSA) for 1 h at room temperature, and then incubated overnight at 4 °C with primary antibodies against target proteins. Primary antibodies used were listed in Table 1. Following primary antibody incubation, samples were incubated with fluorescence dye-conjugated secondary antibodies for 1 h in the dark, washed with PBST, and mounted with a 4′,6-diamidino-2-phenylindole (DAPI)-containing mountant (ProLong Gold Antifade Mountant, ThermoFisher, Waltham, MA, USA, #P36930). Images were then taken with a fluorescence microscope. For THY1-mecherry imaging, HONE1 cells expressing THY1-mecherry conjugate protein were first seeded into glass bottom dishes (Mat Tek, Ashland, MA, USA, #P35GC-1.5-14-C) and then imaged directly.

### 2.4. Co-Immunoprecipitation (co-IP)

The co-IP experiments were performed as previously described [21]. Briefly, cells were seeded into a T175 cell culture flask and cultured until 80% confluency to obtain sufficient input material for the co-IP study. Non-denaturing lysis buffer was used for protein extraction, and the cell lysate was then immunoprecipitated with antibodies (Table 1), with IgG serving as a control.

### 2.5. Western Blot (WB) Analysis

WB analysis was performed as previously described [22]. For the total lysate, cells were lysed in lysis buffer containing 1% of NP40, 250 mM of Tris, 150 mM of NaCl, 0.25% of protease inhibitor, and 1% of phosphatase inhibitor. The lysates were centrifuged, boiled with SDS containing buffer for 10 min for protein denaturation and unfolding, and stored at −20 °C freezer until usage. For the detection of proteins in different cell compartments, lysate was first fractionated with a subcellular proteome extraction kit (Millipore, Burlington, MA, USA, #539790), and then denatured in a similar way for analysis. For the Western blotting (The uncropped blots from this paper’s Figures are shown in Supplementary File S1), an equal amount of protein samples was added to SDS-polyacrylamide gel for resolving. The resolved protein samples were then transferred to PVDF membranes (Millipore, Burlington, MA, USA). The membrane was then blocked by 5% of non-fat milk. After 1 h, the primary antibodies (listed in Table 1) against target proteins were applied and then incubated for 2 h, followed by washing with 5% TBST. Subsequently, HRP-conjugated secondary antibody incubation was conducted, and substrates were added for the detection of the protein. The protein bands were detected by exposure to X-ray films.

### 2.6. Cell Transfection

The THY1 shRNA sequence was based on our previous study with successful knockdown of THY1 [5]. Transfection was then conducted in a similar way for THY1 expression, as described in 4.2. For SRC and PTPN22 knockdown, siRNAs (siTHY1, Dharmacon, Lafayette, CO, USA, #L-015337-00-0005; siSRC, ThermoFisher, Waltham, MA, USA, #s13414; siPTPN22, ThermoFisher #s25225; siCTL, Dharmacon #D-001810-10-20) were transfected into NPC cells with Lipofectamine^®^ 2000 (Invitrogen, Thermo Fisher Scientific, Waltham, MA, USA) as previously described [22].

### 2.7. In Vivo Lung Metastasis Assay

The in vivo lung metastasis assay was conducted on female athymic BALB/c/nu/nu nude mice (6–8 weeks old). The mice were supplied by the Laboratory of Animal Unit of the University of Hong Kong and were kept in the animal house of the Hong Kong Baptist University. The mice were housed in sterile systems with independent ventilation and provided with sufficient sterile water and food. HONE1/HONE1-THY1 were injected via tail vein into mice at a dosage of 5 × 10^5^ cells in 200 μL PBS. Mice were sacrificed at the end of the experiment, and the lungs were stained with H&E staining following a standardized protocol to visualize metastasized tumor cells. Metastasized tumor lobes in lungs were counted manually. For the study of the inhibitory role of KX2-391 on NPC cells, the luciferase labelled NPC HONE1 cells (HONE1-luc) were used. 5 × 10^5^ cells in 200 μL PBS were injected into tail vein of mice. 3 days following the injection of tumor cells, KX2-391 treatment (i.v.) at a dosage of 10 mg/kg, twice per week, was initiated. The treatment schedule refers to previous publications [23,24]. After a treatment period of 35 days, luminescence signals from infiltrated tumor cells were detected with the NightOWL II LB983 in vivo imaging system (Berthold Technologies GmbH & Co, Bad Wildbad, Germany) and mouse lungs were harvested for H&E examination.

### 2.8. Immunohistochemical (IHC) Staining

Lung metastasized HONE1-VA/HONE1-THY1 tumor tissue slides were stained with THY1 primary antibody (1:100 dilution; antibody information in Table 1) for IHC as previously described [4].

### 2.9. TOP/FOP-FLASH Assay

The TOP promoter containing a TCF-binding site and the FOP promoter containing a mutated TCF-binding site were cloned upstream of GFP and packed into lentivirus, transfected into target cells, and selected in a similar way as described in Section 2.2. Fluorescence signals reflecting the activity of Wnt activation were read with a plate reader, and representative images were captured with a fluorescence microscope.

### 2.10. Protein Identification by Mass Spectrometry

Mass spectrometry was performed as previously described [25]. Protein bands excised from SDS-PAGE gels were subjected to reduction with dithiothreitol, alkylation using iodoacetamide, and in-gel digestion with trypsin. Peptide extraction was then performed, followed by the analysis of the peptides using a mass spectrometer (LTQ Velos Dual-Pressure Ion Trap Mass Spectrometer, Thermo Fisher Scientific, Waltham, MA, USA). The obtained tandem mass spectra were subjected to searches in a gene database using the MASCOT search engine (Matrix Science) to identify proteins from the mass spectrometry data.

### 2.11. Invasion Assay

The cell invasion assays were performed as previously described [5]. For the study of the role of SRC and PTPN22, cells treated with siSRC/siPTPN22 or siCTL were seeded into the chamber of a 24-well Matrigel-coated micropore membrane filter with 8 μm pores size (Corning, Somerville, MA, USA, #3442). The bottom 24-well was filled with FBS-containing complete medium, while the upper chamber contains plain medium. For the study of PDGF-BB induced invasion, the upper chamber contained plain medium, while the bottom 24-well contained plain medium plus PDGF-BB. After 36 h incubation at 37 °C, membranes were fixed and stained with crystal violet for visualization.

### 2.12. MTT Assay

NPC cells or immortalized NP69 cells were seeded in 96-well for 24 h. Cells were then treated with KX2-391, bosutinib, and saracatinib at concentrations of 1.9, 5.6, and 16.7 μM for 3 days. For low concentration treatment of KX2-391, 0.94–60 nM of the drug was used to treat the cells for 3 days. After treatment, the original medium was removed, and cells were incubated with a solution of MTT (working concentration 0.5 mg/mL, in 100 μL complete medium) at 37 °C for 3 h. Then, 70% of the medium was carefully removed, DMSO (100 μL) was added, and the plate was shaken for 30 min to solubilize the formazan produced by living cells. The optical densities were measured, and the percentage of inhibition compared to control was calculated.

### 2.13. Statistics

Data are presented as the mean ± standard deviation of ≥3 independent experiments. The difference between control and treatment groups was determined by Student’s *t*-test for two groups or one-way ANOVA for three or more groups, using IBM SPSS version 22.0. *p* < 0.05 was considered to indicate a statistically significant difference.

## 3. Results

### 3.1. THY1 Is Involved in Maintaining Adherens Junctions of NPC Cells

**Figure 1:**
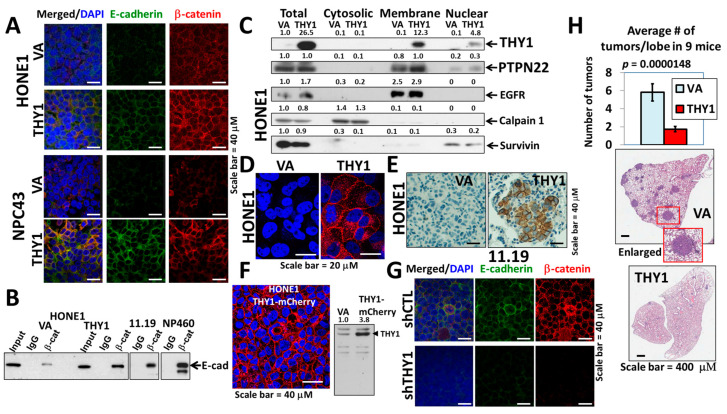
THY1 is involved in maintaining adherens junctions in NPC. (**A**) IF staining of β-catenin and E-cadherin in the HONE1 and NPC43 THY1 transductants. VA, vector-alone. (**B**) Formation of the E-cadherin-β-catenin complex in the THY1-expressing cells. Input stands for total lysate without antibody pulldown. (**C**) Expression of proteins of interest in the cytosol, membrane, and nuclear fractions of THY1 transductants. (**D**) IF staining of THY1 (red) in transplanted THY1 transductants. (**E**) IHC staining of THY1 in transplanted THY1 transductants (small tumor). (**F**) The fluorescent signal of the THY1-mCherry fusion protein in HONE1. Western blot analysis of THY1-mCherry in HONE1 was also shown. (**G**) IF staining of β-catenin and E-cadherin in the THY1 shRNA (shTHY1) knockdown clone of HK11.19. shCTL, control shRNA. (**H**) The results of the lung metastasis assay of the THY1 transductants. Representative H&E staining of a single lobe of a lung from the THY1 and VA transductants.

Our previous studies have shown that THY1 expression is associated with tumor metastasis, and THY1 expression can reduce the invasiveness potential of NPC cells [4,5]. As adherens junctions maintain the integrity of epithelial tissue and loss of adherens junctions contributes to EMT, a process that facilitates the metastasis of epithelial tumor cells, we first studied the impact of THY1 expression on the adherens junctions in NPC cells. The immunofluorescent (IF) staining of E-cadherin and β-catenin (two important components of adherens junctions) showed that adherens junctions were observed in both HONE1 and the EBV-positive NPC43 with the exogenous THY1, while the signals in the vector-alone were very weak (Figure 1A), indicating that the presence of THY1 can help to maintain the adherens junctions in NPC cells. The adherens junction formation was further confirmed by the co-immunoprecipitation (co-IP) analysis of the E-cadherin-β-catenin complex (Figure 1B). In order to ascertain the exogenous THY1 is appropriately expressed, Western blot analysis of subcellular fractionated proteins (Figure 1C), IF (Figure 1D), and IHC staining (Figure 1E) in transplanted THY1 transductants (tumor from Figure 1H), and live imaging of the overexpressed THY1-mcherry fusion protein (Figure 1F) were performed; all these results showed that the exogenous THY1 was primarily localized to the cell membrane, where this protein is supposed to be present. Furthermore, the adherens junctions were also observed in the MCH HK11.19 and the immortalized nasopharyngeal epithelial NP460 cell lines with physiological THY1 expression [4] (Figure 1B,G), and the THY1-knockdown cells of HK11.19 showed reduced signals of the adherens junctions (Figure 1G). When THY1 was constitutively expressed, the numbers of metastatic tumors in the lungs (cells were injected via the tail vein) were significantly decreased by ~5 times (Figure 1H). Taken together, the inhibitory effect of THY1 on NPC metastasis was likely due to the formation of adherens junctions that firmly join the adjacent tumor cells and restrict their mobility when THY1 was present.

### 3.2. THY1 Inhibits Nuclear Translocation of β-Catenin and Inhibits EMT in NPC

**Figure 2:**
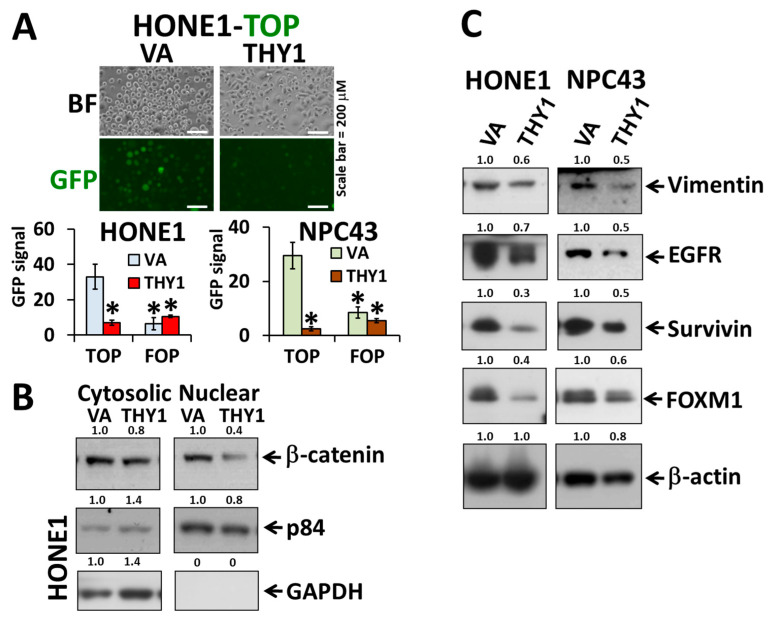
THY1 reduces the level of cytosolic β-catenin and inhibits the Wnt signaling pathway. (**A**) Representative results of TOP/FOP-FLASH assay (top photo) and statistics of the TOP/FOP-FLASH assay in HONE1 and NPC43 cells. Wnt signals are reflected by average GFP intensities in HONE1 and NPC43 with forced expression of THY1. *: *p* < 0.05 compared with VA-TOP; VA, vector-alone. (**B**) Western blot analysis of nuclear and cytosolic β-catenin in THY1 tranductants. (**C**) Western blot analysis of the EMT and Wnt target proteins in THY1-expressing cells.

With the above mentioned finding that THY1 contributes to the formation of tight adherens junctions, we next explored whether it would have any effect on the translocation of β-catenin and the canonical Wnt activity. The TOP/FOP-FLASH assay showed that the canonical Wnt activities were reduced in HONE1 and NPC43 cells, when THY1 was over expressed (Figure 2A). A Western blot of the cytosolic/nuclear fraction showed that the nuclear β-catenin level was significantly reduced (Figure 2B). Such a reduction in canonical Wnt activity is in agreement with the reduction of colony formation potential (self-renewal ability) reported in our earlier study [5], as Wnt signaling promotes the self-renewal potential of NPC cells. The EMT and Wnt target proteins, including vimentin, EGFR, survivin, and FOXM1, were consistently down-regulated in the two NPC cell lines with forced THY1 expression (Figure 2C). As can be seen, THY1 could retain the β-catenin in the cell membrane, prevent the nuclear translocation of β-catenin, and suppress EMT in NPC cells.

### 3.3. THY1 Inhibits SRC Activation in NPC

**Figure 3:**
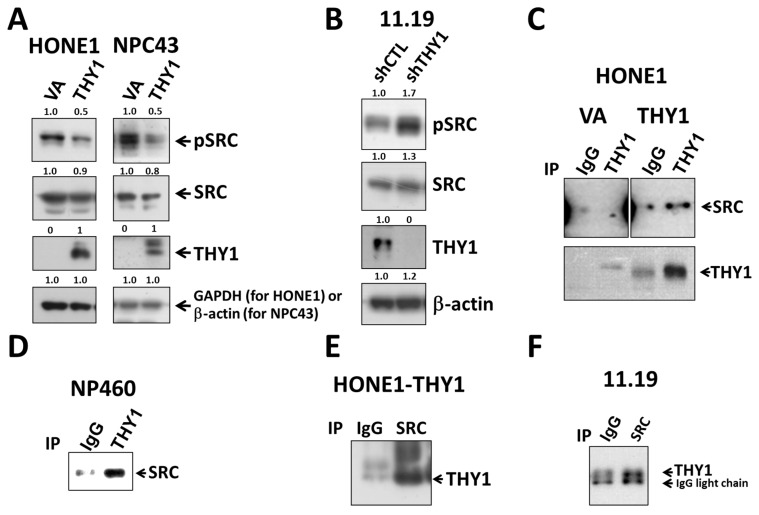
THY1 inhibits SRC activation and is physically associated with SRC in NPCs. Western blot analysis of pSRC (Y419), total SRC, and THY1 in (**A**) THY1-expressing NPC cells and (**B**) THY1-knockdown HK11.19 cells were shown. Co-IP analysis of SRC and THY1 using anti-THY1 was performed in (**C**) HONE1-VA, HONE1-THY1, and (**D**) NP460 cells. Using anti-SRC, co-IP analysis of SRC and THY1 was performed in (**E**) HONE1-THY1 and (**F**) HK11.19 cells.

To elucidate whether the previously reported binding target of THY1, SRC, was involved in the THY1-mediated tight adherens junctions in NPC cells, the phospho-SRC^Y419^ (the active form of SRC) expression in THY1 transductants were studied. Results clearly showed that SRC activation could be suppressed by the presence of THY1 in both HONE1 and NPC43 cell lines (Figure 3A). When THY1 was depleted in HK11.19 cells, the expression of phospho-SRC^Y419^ was increased (Figure 3B). The SRC activities were inversely associated with the THY1 expression in NPC cells. Interestingly, the co-IP assay results showed that THY1 could physically interact with SRC in NPC cells (HONE1, HK11.19) and immortalized NP460 cells (Figure 3C–F), which is in agreement with the observation in other cell types [26,27]. Taken together, these data support the hypothesis that THY1 can inhibit the activation of SRC in NPC cells.

### 3.4. PTPN22 Is Essential for THY1 to Inhibit SRC Activation

**Figure 4:**
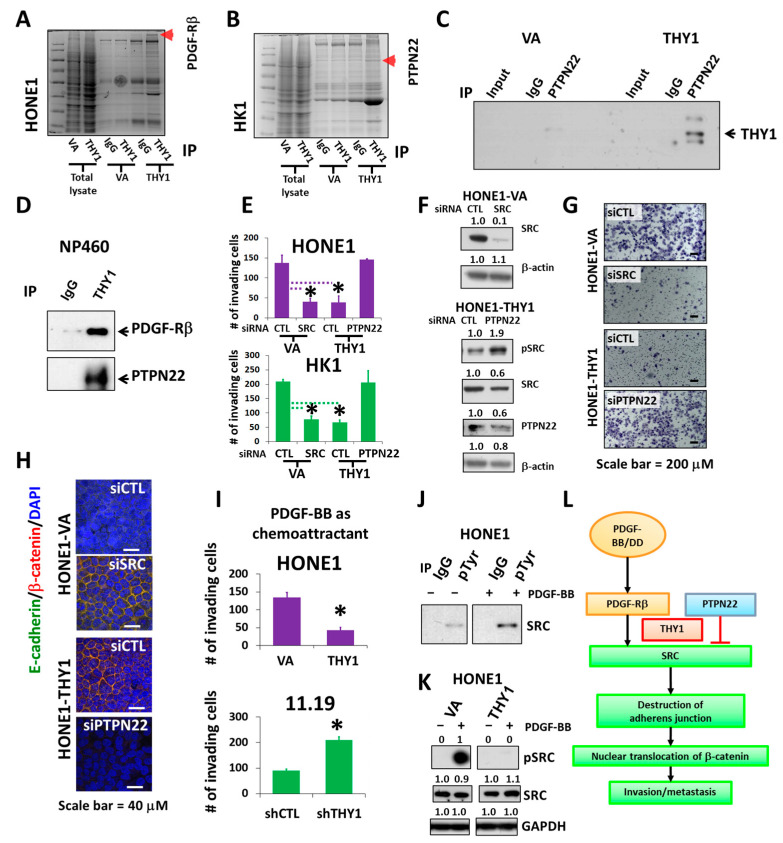
PTPN22 is essential for THY1 to inhibit SRC activation. Coomassie staining of immunoprecipitated and total lysates of cell lines as indicated. Arrows indicate (**A**) PDGF-Rβ and (**B**) PTPN22, identified by mass spectrometry and validated with co-IP. The interaction of THY1 with PDGF-Rβ and PTPN22 were verified with co-IP analysis in HK1 cells in (**C**), and NP460 cells in (**D**). (**E**) Chamber invasion assay for SRC and PTPN22 siRNA depletion in HONE1-VA, HONE1-THY1, HK1-VA, and HK1-THY1 cells. *: *p* < 0.05. (**F**) Western blot analysis of SRC and PTPN22 in siRNA knockdown cells. (**G**) Representative images of the invaded HONE1-VA and HONE1-THY1 cells with SRC or PTPN22 knockdown. (**H**) IF staining of adherens junctions (merged images of green E-cadherin and red β-catenin) for cell lines described in Figure 4G. (**I**) Chamber invasion assay using PDGF-BB as a chemoattractant for HONE1-VA and HONE1-THY1 cells, HK11.19 and THY1-knockdown HK11.19 cells. *: *p* < 0.05. (**J**) Co-IP analysis of phospho-SRC in the presence of PDGF-BB in HONE1. (**K**) Western blot analysis of pSRC (Y419) and total SRC in THY1-expressing cells in response to PDGF-BB. (**L**) Schematic illustration of how THY1 can regulate EMT and SRC activity through the interaction with PTPN22 and PDGF-Rβ.

In order to study the downstream mechanism(s) of THY1, proteomic profiling by mass spectrometry was performed to identify its candidate binding partners (Figure 4A,B). PDGF-Rβ and PTPN22 were found to be candidate binding proteins of THY1, and the interaction was subsequently validated by the co-IP analyses in NPC (with forced THY1 expression) and NP460 cells (Figure 4C,D).

PTPN22 is a protein tyrosine phosphatase that is primarily expressed in the lymphoid tissues and is involved in modulation of the T cell receptor (TCR), B cell receptor, and toll-like receptor pathways, etc. Numbers of SFKs are indeed the substrates of PTPN22. PTPN22 by itself can efficiently dephosphorylate SFKs at activating tyrosine residues in the PTK domains and subsequently suppress the SFK-mediated TCR signals [28,29,30]. Interacting with PTPN22 might be important for THY1 to suppress SRC in NPCs. The PTPN22 siRNA silencing results showed that the tumor invasion inhibitory activity of THY1 was lost in HONE1-THY1 and HK1-THY1 cells when PTPN22 was depleted (Figure 4E–G). The SRC siRNA silencing could reduce the invasion of HONE1 and HK1 cells, which are comparable to the inhibitory effects of THY1 (Figure 4E–G). Furthermore, the SRC knockdown had restored the adherens junctions in HONE1 cells (Figure 4H). In addition, maintenance of adherens junctions by THY1 was lost in the HONE1-THY1 cells when PTPN22 is knocked down (Figure 4H), which could be due to the increased active SRC expression. Interestingly, it has been demonstrated that once PTPN22 was dissociated from CSK in T cells, PTPN22 was recruited to the lipid rafts of the plasma membrane to down-modulate the T cell receptor signaling by dephosphorylating the SFK LCK [31]. Our subcellular fractionation analysis has clearly shown that nearly all the PTPN22 protein was located in the membrane fraction (Figure 1C). Those data suggest that THY1 can interact with PTPN22 in the lipid raft to reduce SRC activity, maintain adherens junctions, and suppress the invasiveness of NPC cells.

PDGF-Rβ is a transmembrane receptor tyrosine kinase; increased expression of PDGF-Rs on tumor cells is associated with EMT. Epithelial tumor cells undergoing EMT become responsive to stimulation by the ligand PDGF [32] and PDGF/PFGF-R can activate SRC and the subsequent tumor formation and metastasis [33]. The role of PDGF-Rβ in SRC activation and THY1-mediated suppression of tumor invasion was examined. When PDGF-BB, the ligand of PDGF-Rβ (50 ng/mL), was used as a chemoattractant for the invasion assay, the results showed that the PDGF-BB-induced cell invasion was suppressed, when THY1 was present (in both HONE1-THY1 and HK11.19 cells) (Figure 4I). In addition, PDGF-BB (50 ng/mL) could increase the phospho-SRC level (Figure 4J). The SRC activation by PDGF-BB was further confirmed by the induction of a high level of phospho-SRC^Y419^ in HONE1 cells and could be completely suppressed, when THY1 is expressed (Figure 4K). Thus, THY1 could suppress the PDGF-Rβ/SRC signaling activities in NPC, and such suppression is associated with the suppressed invasion capability of THY1.

Taken together, the above data suggested that THY1 can maintain the adherens junctions in NPC cells by the presence of PTPN22 to counteract SRC activation, which can be induced by PDGF-Rβ, and this is how NPC tumor invasion and metastasis can be inhibited by THY1. A schematic diagram of this proposed model is presented in Figure 4L.

### 3.5. Inhibition of SRC Suppresses Metastasis of NPC Cells

**Figure 5:**
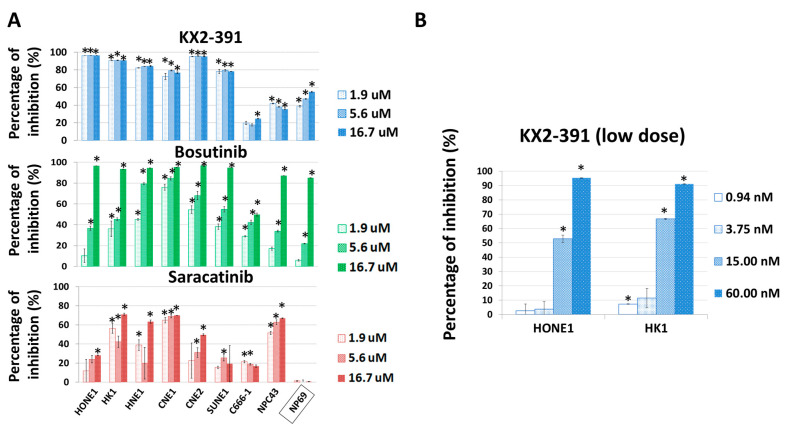
SRC inhibitors suppress the growth of NPC cells. (**A**) The in vitro growth inhibitory effects of KX2-391, bosutinib, and saracatinib were tested with a panel of NPC cell lines and the immortalized NP cell line NP69. The numbers of viable cells were detected by the MTT assay after treatments for 3 days. The relative inhibition rate was compared with the corresponding vehicle control. (**B**) The effects of low dosages of the SRC inhibitor KX2-391 on the in vitro growth of NPC cells. Four concentrations were used. The numbers of viable cells were detected by the MTT assay after treatments for 3 days. The relative inhibition rates were compared with their corresponding vehicle controls. *: *p* < 0.05.

**Figure 6:**
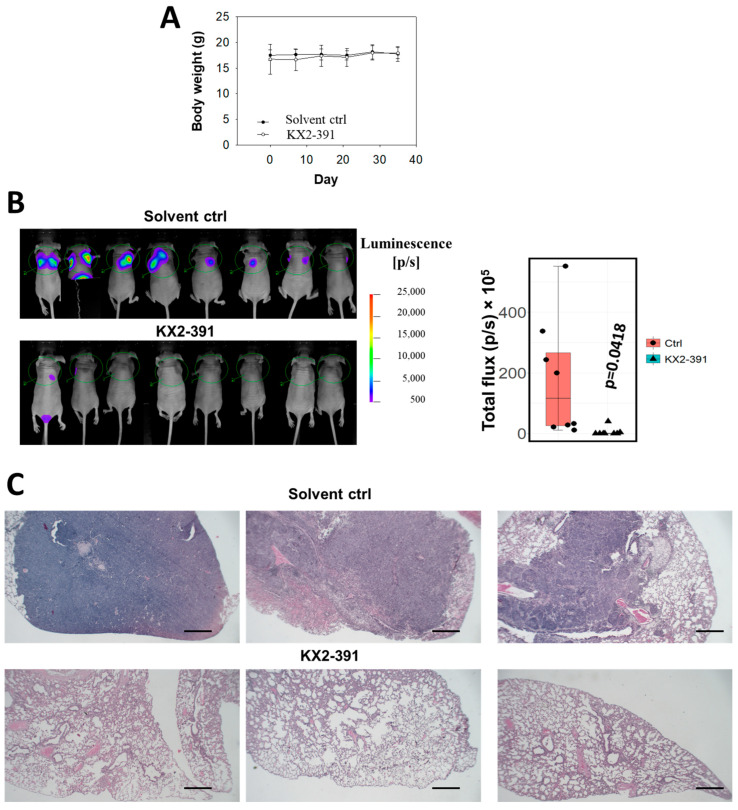
KX2-391 inhibits the lung metastasis of NPC cells. (**A**) The body weight of the mice in the treatment with KX2-391 was analyzed. (**B**) The infiltrated HONE1 cells in the lungs were reflected by the bioluminescence signals in the nude mice (*n* = 8). The luminescence readings from all the mice were presented as a box plot, and each data point was shown. (**C**) Representative H&E staining of the mouse lungs (Scale bar, 2 mm).

From the above findings, it is likely that the inactivation of SRC by siRNA or THY1 expression is sufficient to suppress NPC invasion and metastasis. Those results arouse our interest in studying the antagonistic agents of SRC to control NPC progression. Three SRC inhibitors, KX2-391, bosutinib, and saracatinib, which had undergone clinical trials for other cancer types [34], were chosen for testing of their anti-tumor effects on a panel of eight NPC cell lines. The immortalized nasopharyngeal epithelial (NP) NP69 cell line served as a non-tumorigenic control. The cell viability was detected by the MTT assay. Those cell lines were treated with different dosages of those three drugs separately for three days, and KX2-391 showed the highest growth inhibition (Figure 5A). We found that the responses of each cell line to the three drugs were quite diverse. KX2-391 was quite effective for reducing the cell growth of the NPC cell lines (70–95% inhibition with 1.9 µM), while saracatinib showed more tumor-specific effects, as it could selectively reduce the tumor growth of all eight NPC cell lines and had no significant effect on NP69 with all three concentrations tested (1.9–16.7 µM). When the drug concentrations of KX2-391 were further scaled down to a nanomolar range (0.94–60 nM), it was found that 15 nM of the drug was sufficient to reduce the viable cell numbers of HK1 and HONE1 cells by 67% and 53%, respectively (Figure 5B). Based on the high potency of KX2-391 in suppressing the growth of NPC cells, the in vivo effect on the metastasis of NPC cells was investigated. Luciferase reporter HONE1 cells were injected into nude mice via tail vein, followed by treatment with 10 mg/kg of KX2-391 or solvent control at a frequency of twice per week for 5 weeks. The average body weights of the two groups of mice were similar throughout the experiment period (Figure 6A), suggesting that such a dosage of KX2-391 is well tolerated by the mice. The bioluminescence signals emitted by the metastatic lung tumors in the KX2-391 treatment group were drastically reduced when compared to the solvent control group. The average signal was 32-fold higher in the control group of mice than in the treatment group (*n* = 8) (Figure 6B). H&E staining of mouse lungs was used to validate the bioluminescence results, and the results (Figure 6C) showed that in the control group, lung infiltration of NPC cancer cells was much more severe than in the treatment group. It can be seen that inhibition of SRC activity could efficiently suppress the metastasis of NPC cells in this animal model.

## 4. Discussion

Metastasis remains one of the biggest challenges in the management of NPC, and hyperactivity of SRC has been found to be associated with poor prognosis and to induce EMT and subsequently enhance the metastasis potential of NPC cells [16,17]. To our knowledge, the approach to controlling NPC progression by inhibiting SRC has not been well explored. In the present study, we have included three popular SRC inhibitors that had entered clinical trials for other diseases [34]. Those drugs were tested with a panel of NPC cell lines, and KX2-391 was identified as a potent inhibitor to suppress NPC cell growth. Subsequent in vivo studies showed that KX2-391 can inhibit the lung metastatic ability of NPC cells (with mean luminescence signals reduced by ~32-fold). These results demonstrated the drug efficacy of KX2-391 in inhibiting both cell growth and metastasis of NPC cells, indicating that SRC inhibition may be a promising approach to control the progression of NPC.

A recent study has shown that THY1 expressed on mouse basal cells can interact with integrin β1 to suppress its downstream activation of SRC, and loss of THY1 expression is associated with activation of SRC, dissociation of adherens junctions, and enhanced epidermal wound repair [35]. That study suggested that THY1 can play a role in cell adhesion/migration via modulation of SRC activity in the skin, which is in agreement with our current finding. THY1 has a context-dependent role in cancer. In ovarian adenocarcinoma patients, THY1 expression is reduced, and THY1 expression is specifically found in the non-tumorigenic cell clones, but not in the tumorigenic ones [36,37]. In childhood cancer neuroblastoma, low/missing THY1 is associated with poor survival, and patients with low/missing THY1 expression were found to have a higher relapse rate [38,39]. We had previously identified THY1 as a tumor suppressor in NPC [4,5]. On the other hand, THY1 expression was found to be significantly higher in esophageal squamous carcinoma cells (ESCC) when compared with normal or premalignant surrounding tissues and is strongly correlated with lymph node metastasis [40]. Upregulation of THY1 was reported for lung cancer, and such high expression is negatively correlated with survival time [41,42]. Hepatocellular carcinoma tumors were reported to express a high level of THY1, and such high expression of THY1 is associated with poor prognosis [43,44,45]. As can be seen, such a dual role of THY1 is cell context dependent and its interaction with different signaling molecules might explain the various functions in different cancers. For example, Barker and Hagood suggested that THY1 can participate in the trafficking and partitioning of signaling molecules at the lipid rafts and may act alternately to sequester or traffic signaling molecules, resulting in either inhibition or activation of downstream signaling, such as SFKs, in different cell contexts [46]. Indeed, our results clearly showed that SRC activity was inhibited by THY1 in NPC, which is in accordance with the inhibitory role of THY1 in SRC activity in fibroblasts [10], basal cells [35], and neuron cells [47]. Other studies reported that the presence of THY1 is essential for activation of SRC by thrombospondin/hep I [48,49]. From those studies, it can be seen that the effect of THY1 on SRC activity is cell context dependent. Our study demonstrated that THY1 could inhibit the activity of SRC, and that can resulted in the maintenance of adherens junctions and suppression of tumor metastasis in NPC for the first time.

THY1 was first described as a lymphocyte marker in 1964 [50], and over the past 60 years, an increasing body of studies have been conducted to determine its roles in multiple physiological conditions. Yet the exact signaling mechanisms of THY1 remain enigmatic [46]. A study by Maldonado et al. had shed some light on the importance of the function of THY1 [47]. On the cell membrane of rat neuronal cells, THY1 can form nanoclusters, which dynamically associates with or disassociate from C-terminal SRC kinase (CSK)-binding protein (CBP) in lipid rafts. When integrin α_V_β_3_ on the adjacent astrocyte binds to THY1 on the neuron, the binding reduces the mobility of THY1 and causes the formation of large clusters of the THY1-CBP complex. This complex subsequently recruits CSK, which phosphorylates SRC on Y527 (rat) and inhibits its activation. From that study, it can be seen that the interaction of THY1 with other membrane molecules is crucial for its function. To our surprise, although PDGF-R and SRC are well-known oncogenic proteins, their functional roles in NPC are not well defined. As can be seen, the results of this study have opened up a new window to determine if inhibition of PDGF-Rβ/SRC signaling is a potential therapeutic strategy for controlling NPC progression. It would be interesting to explore the role of THY1 in the regionally associated malignancy arising from the nasal septum [51]. THY1 can negatively regulate PDGF-Rβ/SRC signaling, and this was not known before this study. Moreover, we have also demonstrated the novel function of PTPN22; the presence of PTPN22 is essential for THY1 to suppress the EMT phenotype in NPC cells. Yet it should be noted that our in vivo study using the lung metastatic mouse model had some limitations. Due to the lack of an orthotopic metastasis model for NPCs, EMT might not be accurately reflected by the current metastasis model. This study demonstrated the possibility of using PDGF-Rβ/SRC inhibitors for the management of metastatic NPCs. Indeed, the SRC inhibitors dasatinib and bosutinib have been approved for clinical usage in the treatment of chronic myeloid leukemia [52,53], and KX2-391 has been approved for the treatment of actinic keratosis and for the prevention of the development of actinic keratosis into squamous cell carcinoma [54]. Our results on SRC inhibition can provide more therapy options to clinicians who can use those SRC inhibitors to treat advanced NPC cases with metastasis. On the other hand, PDGF-Rβ inhibitors such as ripretinib [55] and sorafenib [56] have been approved for the treatment of gastrointestinal stromal tumor and renal cell carcinoma, respectively. It is worthwhile to investigate the clinical application potential of those PDGF-Rβ inhibitors in treating metastatic NPCs.

## 5. Conclusions

The current study clearly showed that THY1 can inhibit the NPC tumor invasion by the maintenance of adherens junctions, so the EMT in the tumor cells is suppressed by the presence of THY1. The maintenance of adherens junctions by THY1 was subsequently found to be associated with regulation of the level of nuclear β-catenin, and THY1 was found to inhibit SRC activation in NPC. With proteomic profiling of the THY1-interacting partners, two novel THY1 binding proteins, PDGF-Rβ and PTPN22, have been identified to be associated with the maintenance of the adherens junctions. PTPN22 is essential for THY1 to inhibit SRC activation, which can be induced by PDGF-Rβ, and that in turn will support the formation of the adherens junctions. As PTPN22 is a tyrosine phosphatase and THY1 contains no enzymatic activity, their interaction could be important for THY1 to dephosphorylate SRC in NPC cells. The formation of the THY1-PTPN22-PDGF-Rβ-SRC complex warrants further study in order to understand the regulatory role of THY1 in PDGF-Rβ-induced SRC activity. This study also indicates the functional role of the oncogenic molecules PDGF-Rβ and SRC in NPC progression. These results will suggest that PDGF-Rβ and SRC can be used as drug targets for suppressing NPC metastasis.

## Figures and Tables

**Table 1:** List of antibodies used in this study.

Antibody	Manufacture	Catalog Number	Application	Host Species
Calpain 1	Santa Cruz	sc-58323	WB	Mouse
E-cadherin	Cell Signaling	14472	WB	Mouse
EGFR	Cell Signaling	2232	WB	Rabbit
FOXM1	Cell Signaling	5436	WB	Rabbit
GAPDH	GeneTex	GTX627408	WB	Mouse
IgG	Cell Signaling	3900S	IP	Rabbit
IgG	Cell Signaling	5415	IP	Mouse
p84	Abcam	ab131268	WB	Rabbit
PDGF-Rβ	Cell Signaling	3169	WB	Rabbit
PTPN22	Cell Signaling	14693	WB, IP	Rabbit
p-Tyr	Cell Signaling	9411	IP	Mouse
SRC	Cell Signaling	2123	WB, IP	Rabbit
phospho-SRC (Y416/419)	Cell Signaling	6943	WB	Rabbit
Survivin	Cell Signaling	2808	WB	Rabbit
THY1	Cell Signaling	13801	WB, IHC	Rabbit
THY1	Abcam	ab133350	WB, IHC, IF	Rabbit
THY1	Cell Signaling	9798	WB, IP	Rabbit
Vimentin	BD Bioscience	550513	WB	Mouse
β-actin	Sigma	A5441	WB	Mouse
β-catenin	Cell signaling	8480	WB, IF, IP	Rabbit

## Data Availability

All data were included in this article and the Supplementary File.

## Supplementary Materials

The following supporting information can be downloaded at: https://www.mdpi.com/article/10.3390/cancers15072189/s1. Supplementary File S1: Remarks on the original WB blot.
