# Supplementary material for: THY1 (CD90) Maintains the Adherens Junctions in Nasopharyngeal Carcinoma via Inhibition of SRC Activation
Source: Cancers (Basel). 2023 Apr 6;15(7):2189. doi: 10.3390/cancers15072189 (PMC10093038; doi:10.3390/cancers15072189)
Supplement: Supplementary file 1 [file cancers-15-02189-s001.zip › cancers-2275690-supplementary.pdf]

Supplementary File S1: Remarks on the original WB blot.

1. **On the molecular weight marker:**
   When performing western-blot, we were using PageRuler Prestained Protein Ladder from ThermoFisher (Cat_No.: 26616) as protein size standard. All the signal of western-blot were captured through traditional X-ray film exposure. So, the protein molecular weight marker, which did not have chemiluminescence property by itself, could not be visualized on the X-ray film. Protein molecular weight markers were marked on the X-ray film by hand (by overlapping the exposed X-ray film onto the original membrane, and mark down the position of the ladder).In this supplementary file, the hand-marked ladder positions were high lighted with **red solid-line rectangles.**

2. The bands shown in the manuscript were high-lighted with **dashed-line red rectangles**.

3. **On the “full blot” requirement:**
   During the western blot process, blots were already trimmed to appropriate size for the target protein. Therefore, the WB pictures inside the WB supplementary file represent the original western blot result of the full-blot.

Fig 1B

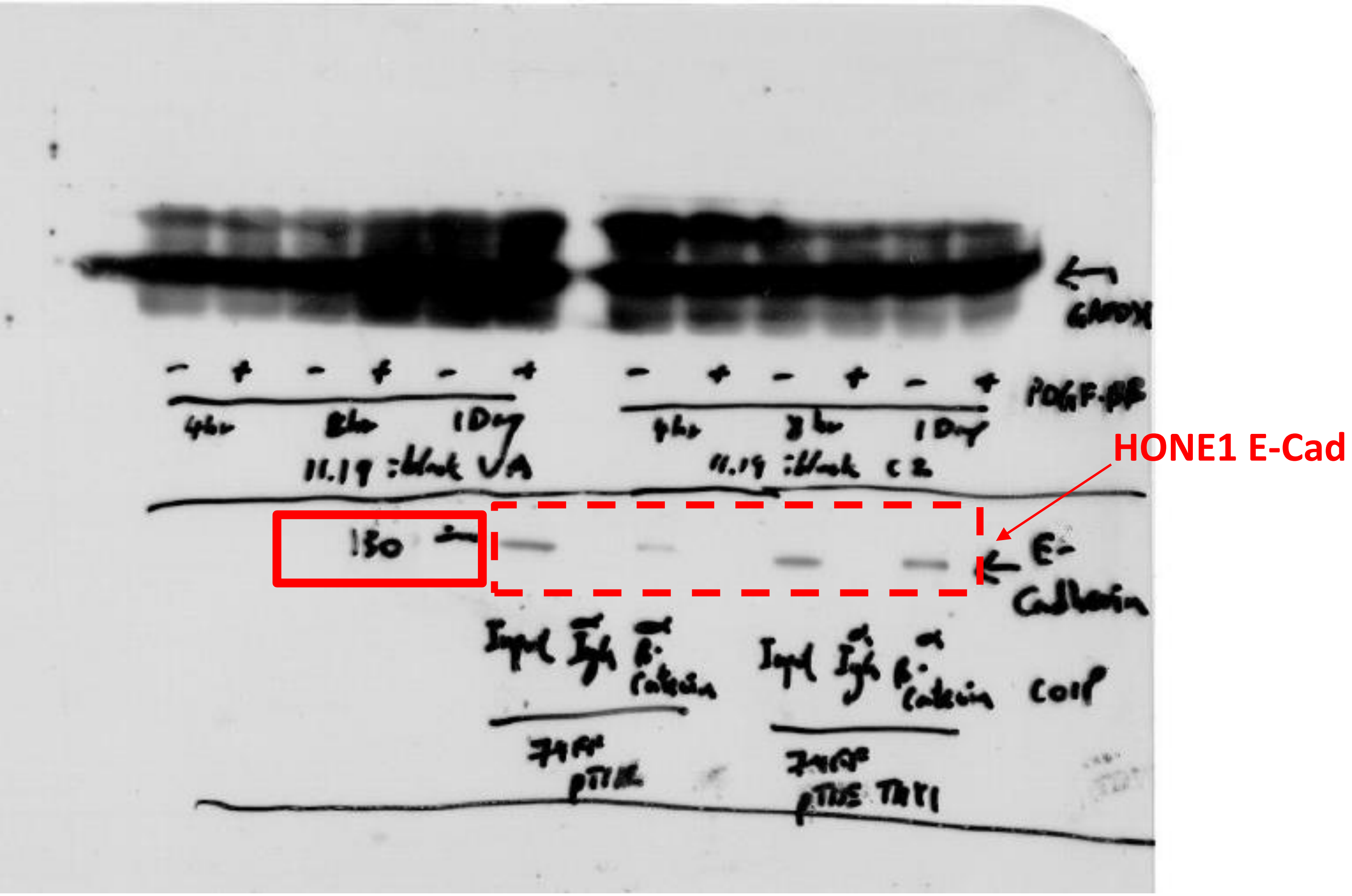

Fig 1B

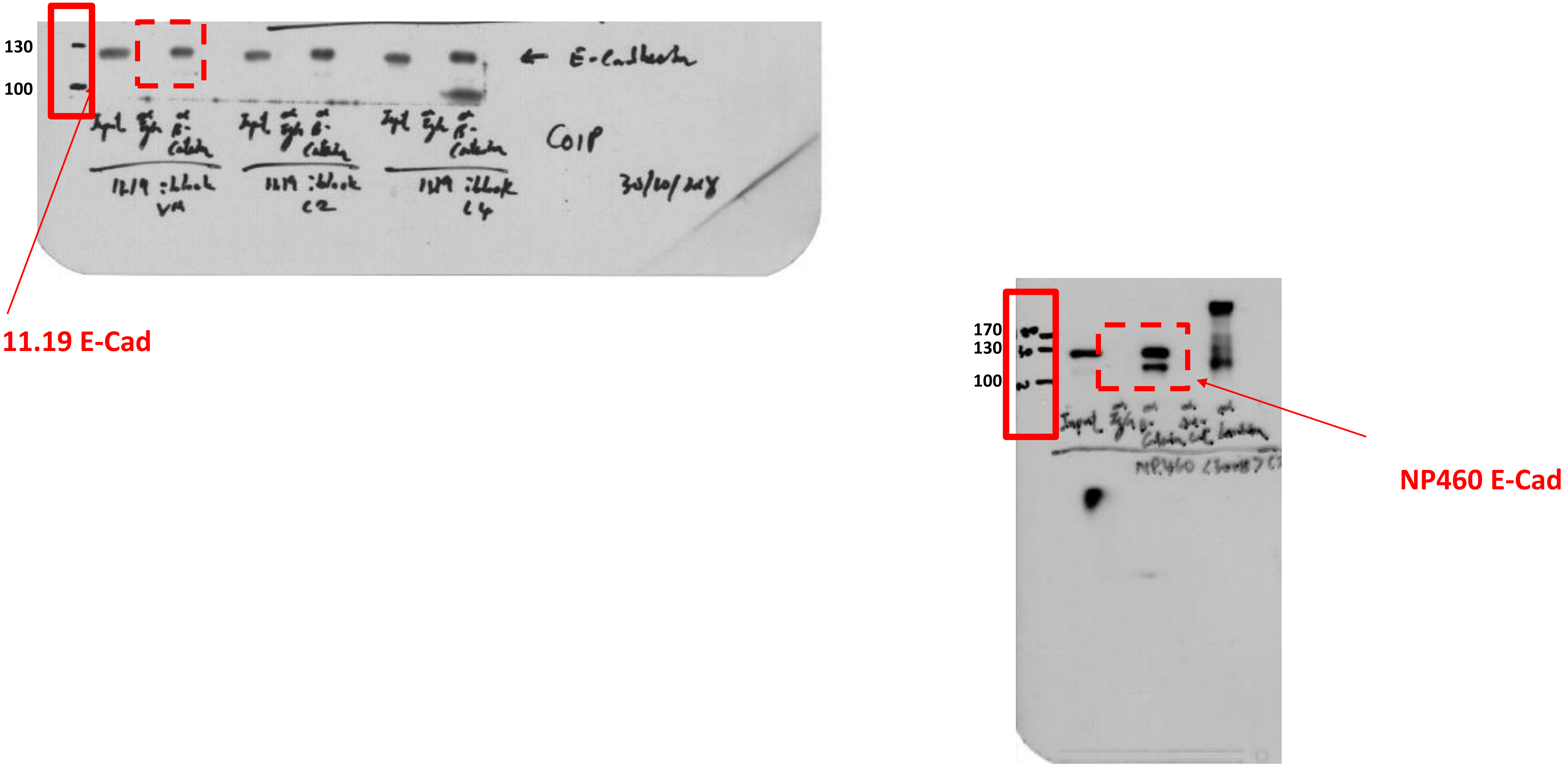

Fig 1C

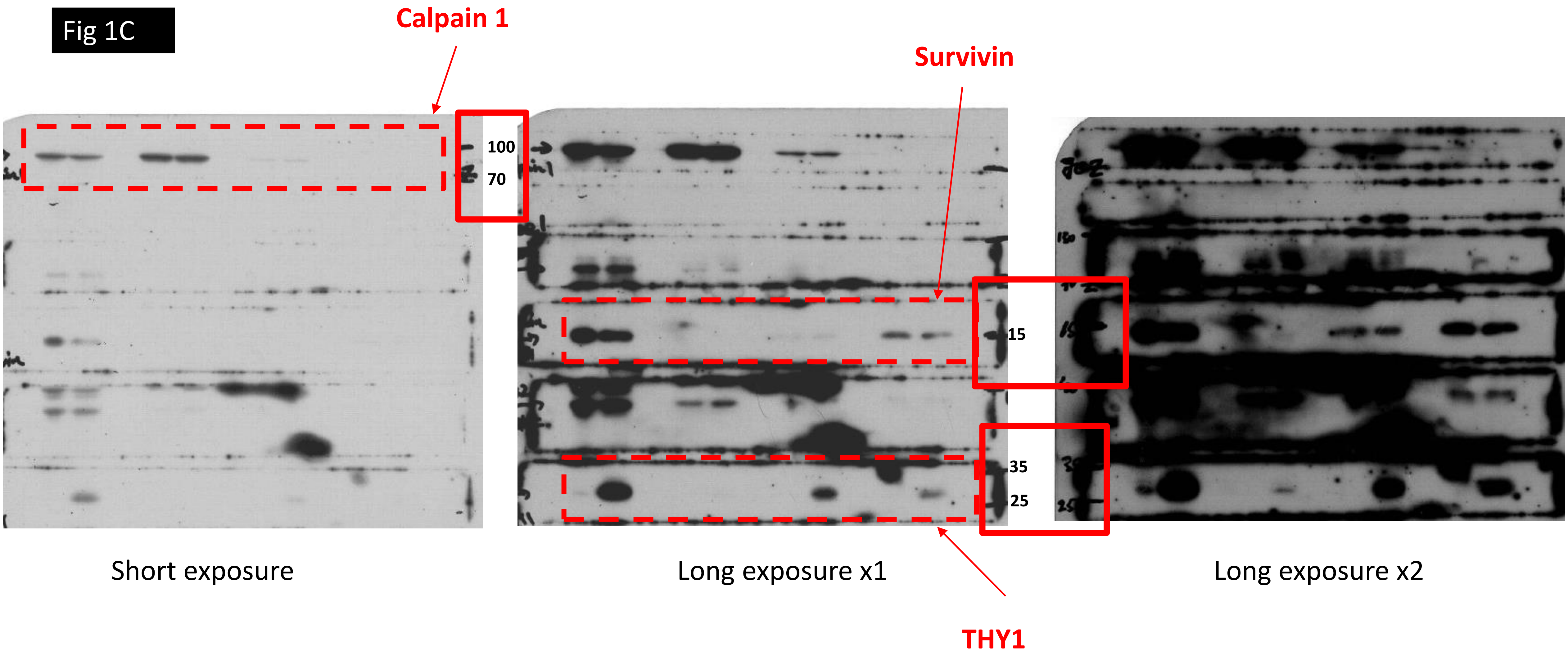

Short exposure

Long exposure x1

Long exposure x2

Fig 1C

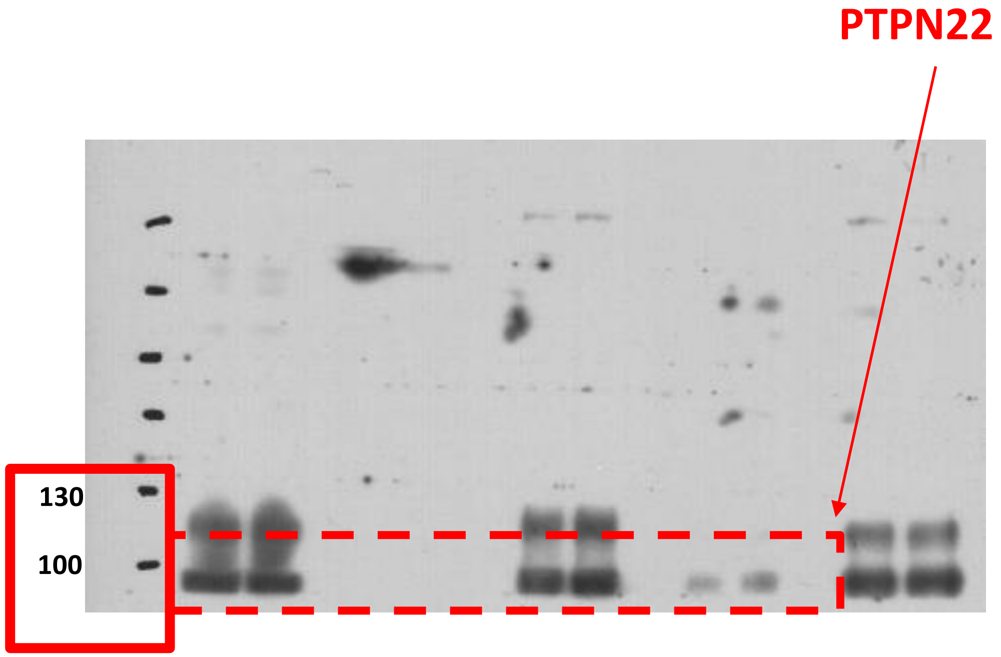

Short exposure

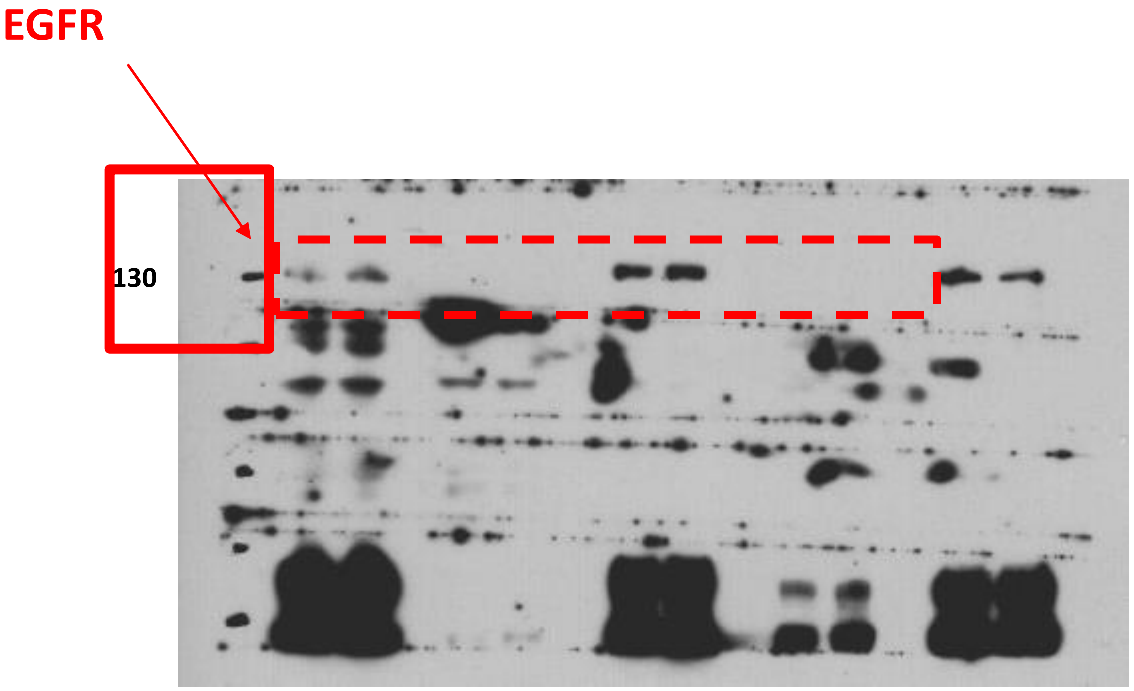

Long exposure x1

Fig 1F

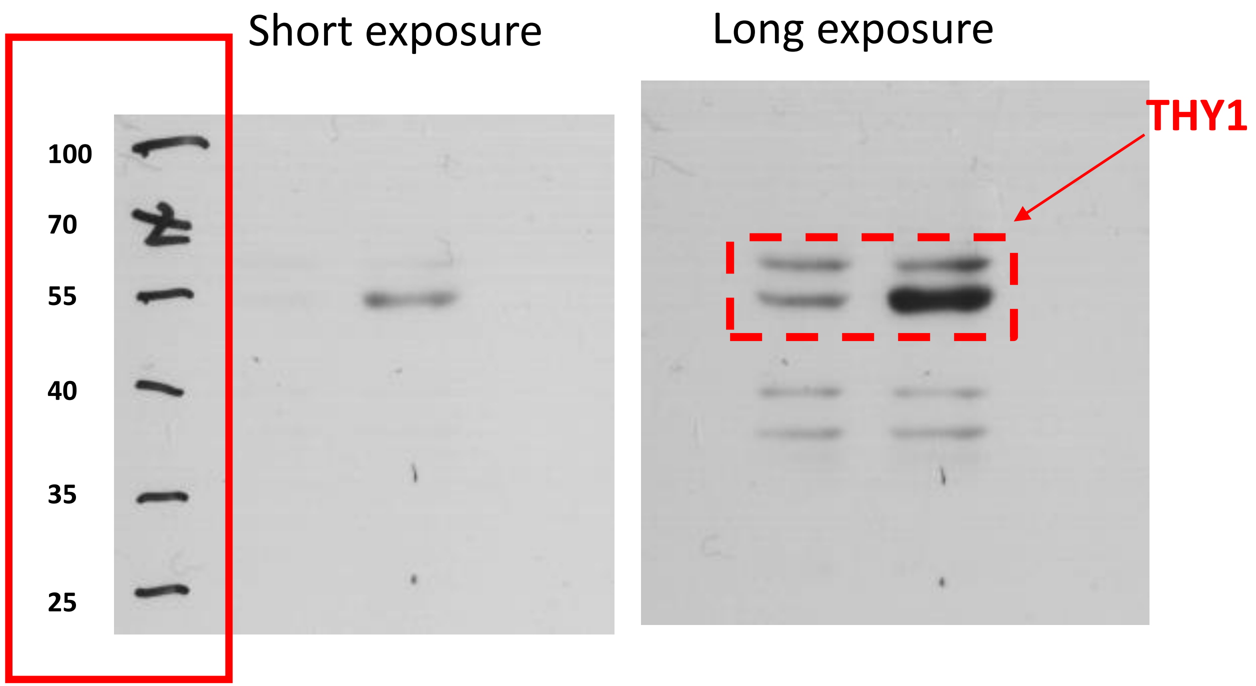

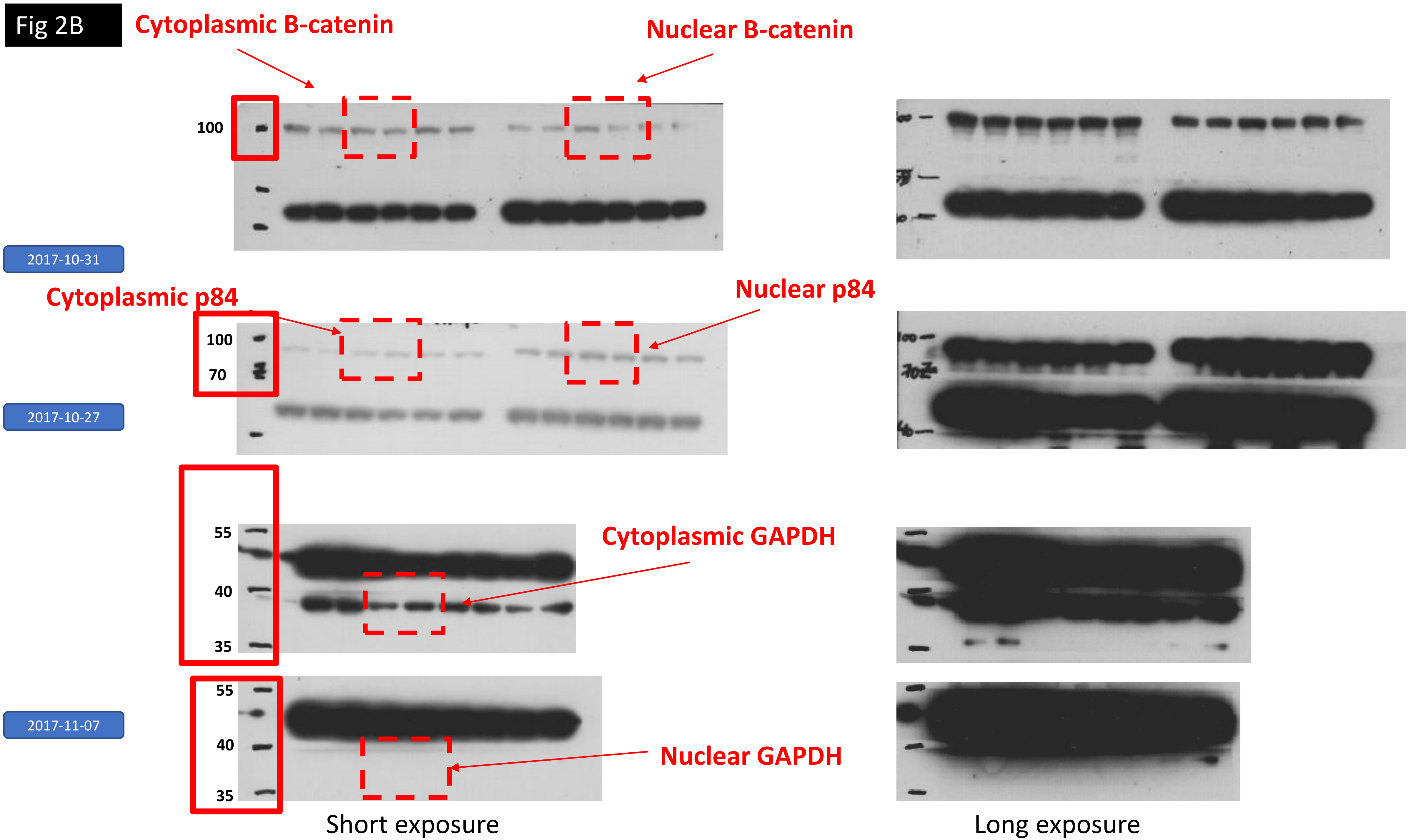

Fig 2B
Cytoplasmic B-catenin
Nuclear B-catenin
100
2017-10-31
Cytoplasmic p84
Nuclear p84
100
70
2017-10-27
55
40
35
Cytoplasmic GAPDH
55
40
35
2017-11-07
Nuclear GAPDH
Short exposure
Long exposure

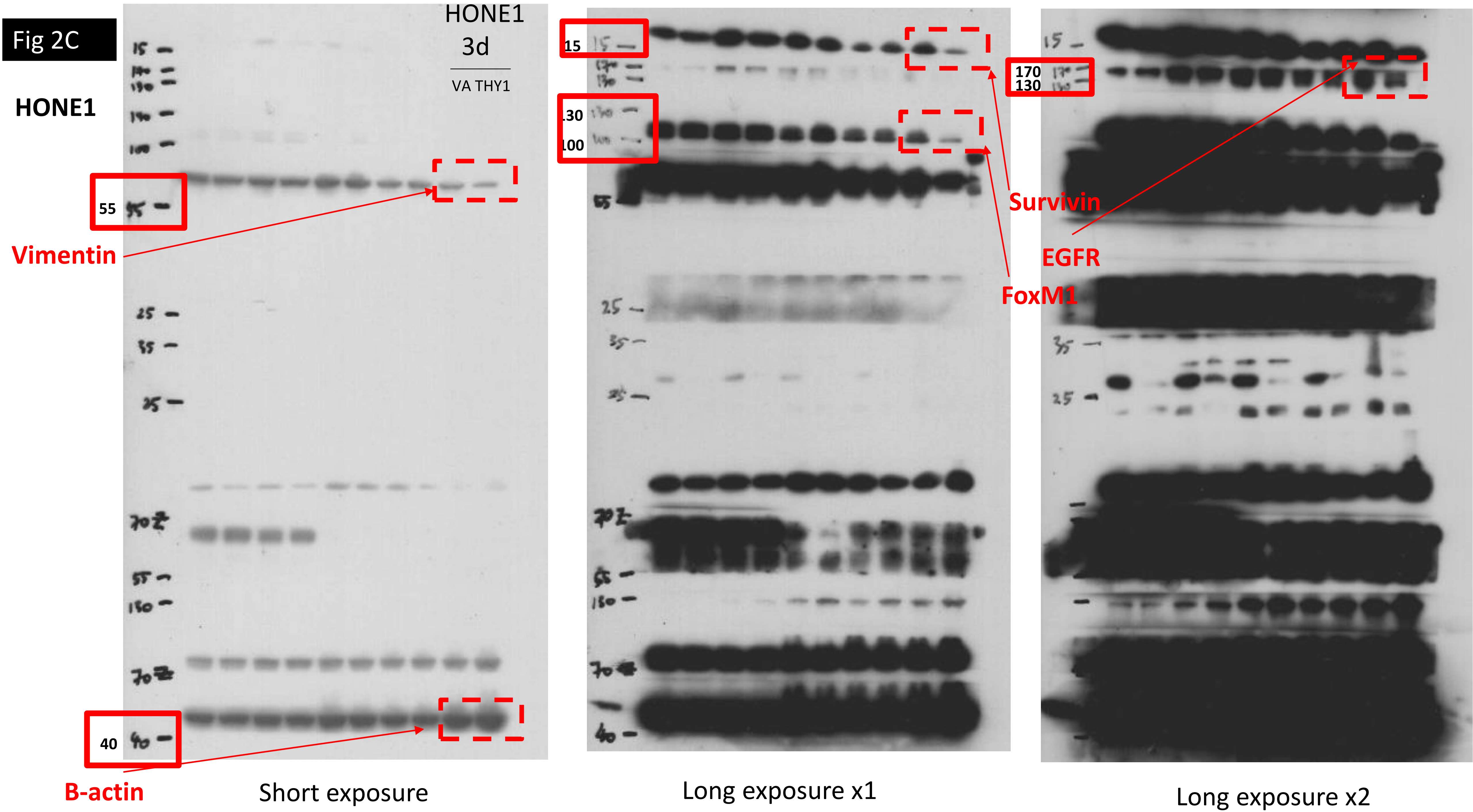
Fig 2C
HONE1
HONE1
3d
VA THY1
55
Vimentin
40
B-actin
Short exposure
15
130
100
Survivin
FoxM1
Long exposure x1
170
130
EGFR
Long exposure x2

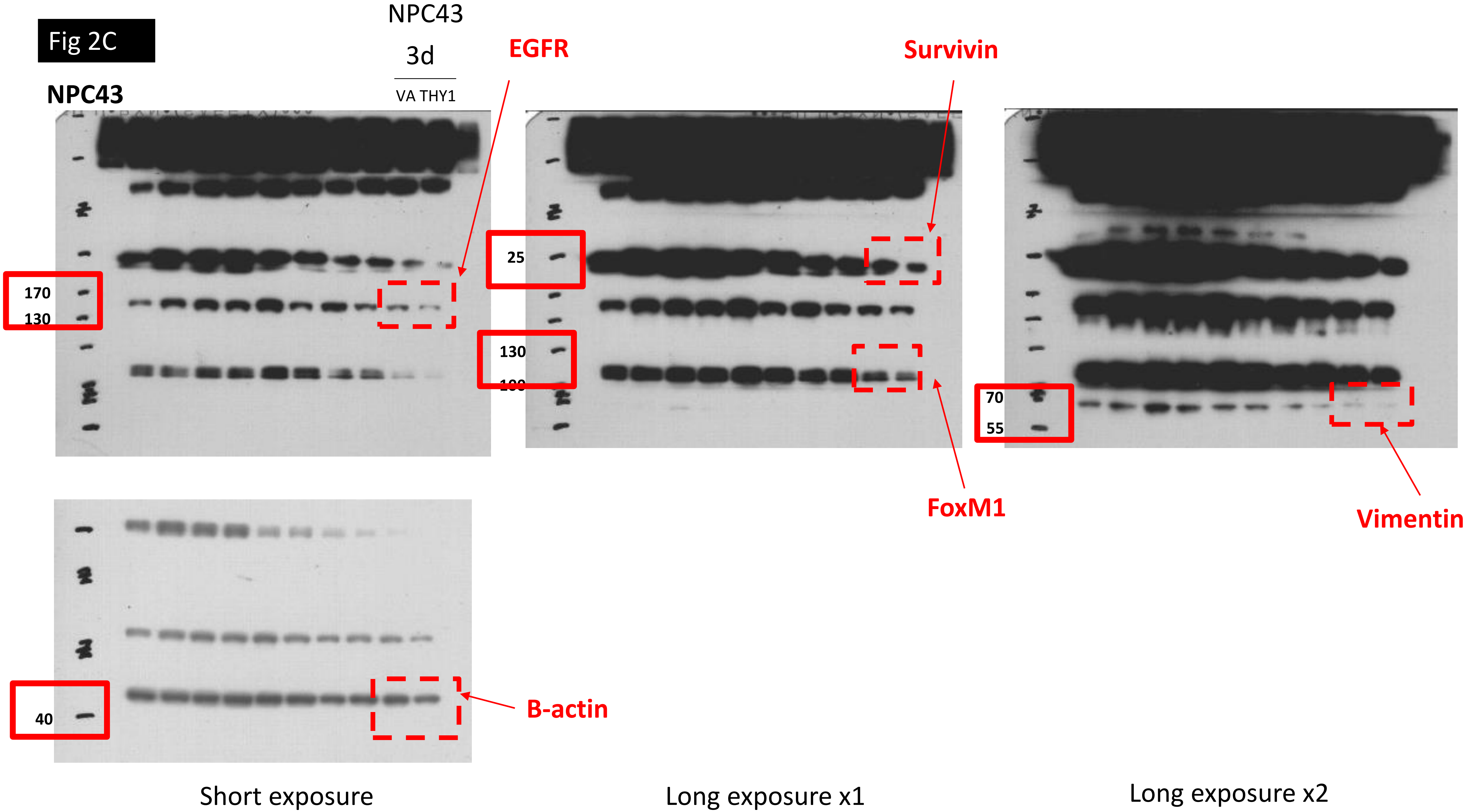
Fig 2C
NPC43
NPC43
3d
VA THY1
EGFR
Survivin
170
130
25
130
100
70
55
FoxM1
Vimentin
40
B-actin
Short exposure
Long exposure x1
Long exposure x2

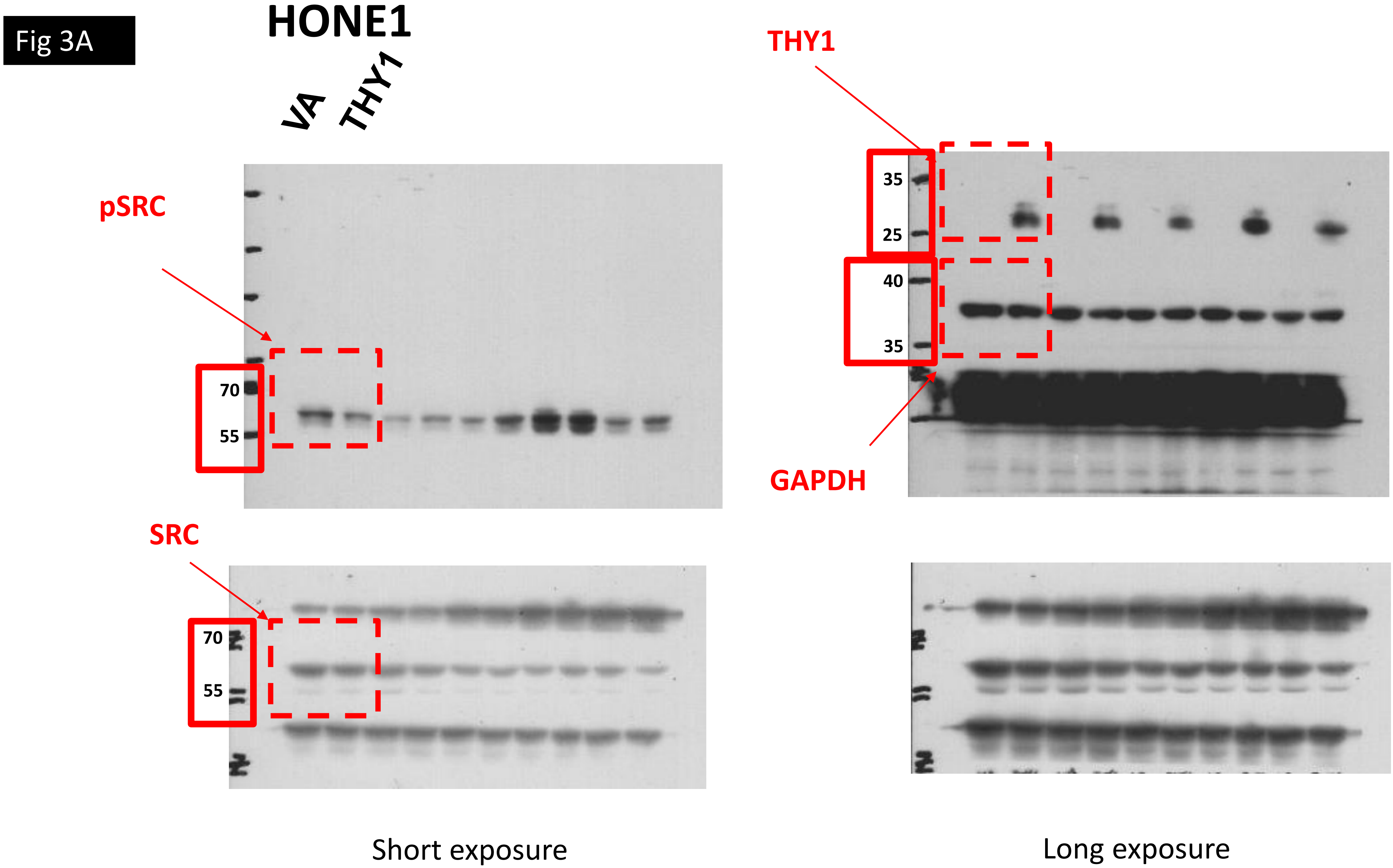
Fig 3A
HONE1
VA
THY1
pSRC
70
55
SRC
70
55
THY1
35
25
40
35
GAPDH
Short exposure
Long exposure

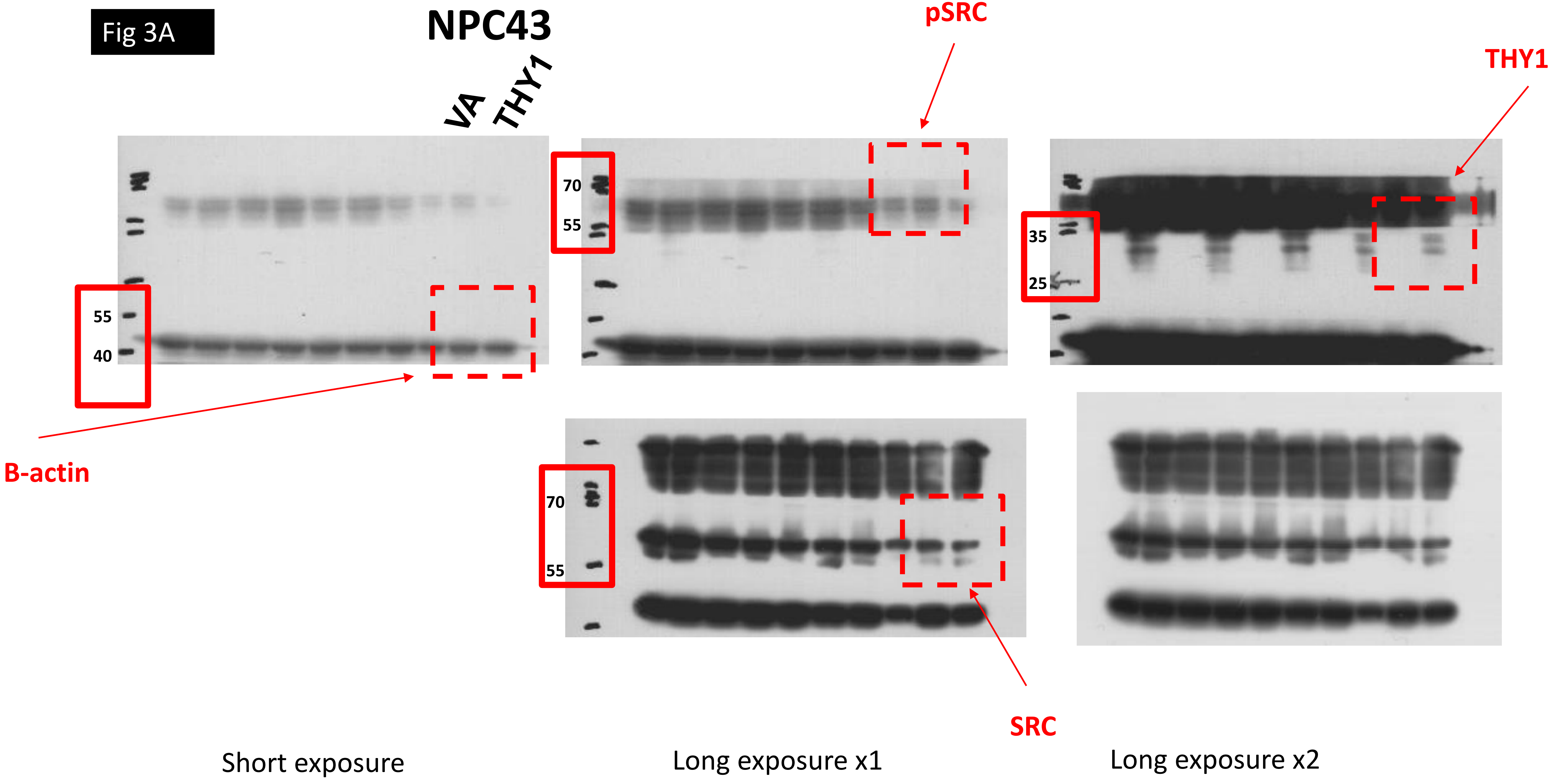
Fig 3A
NPC43
VA
THY1
pSRC
THY1
70
55
55
40
35
25
70
55
B-actin
SRC
Short exposure
Long exposure x1
Long exposure x2

Fig 3B

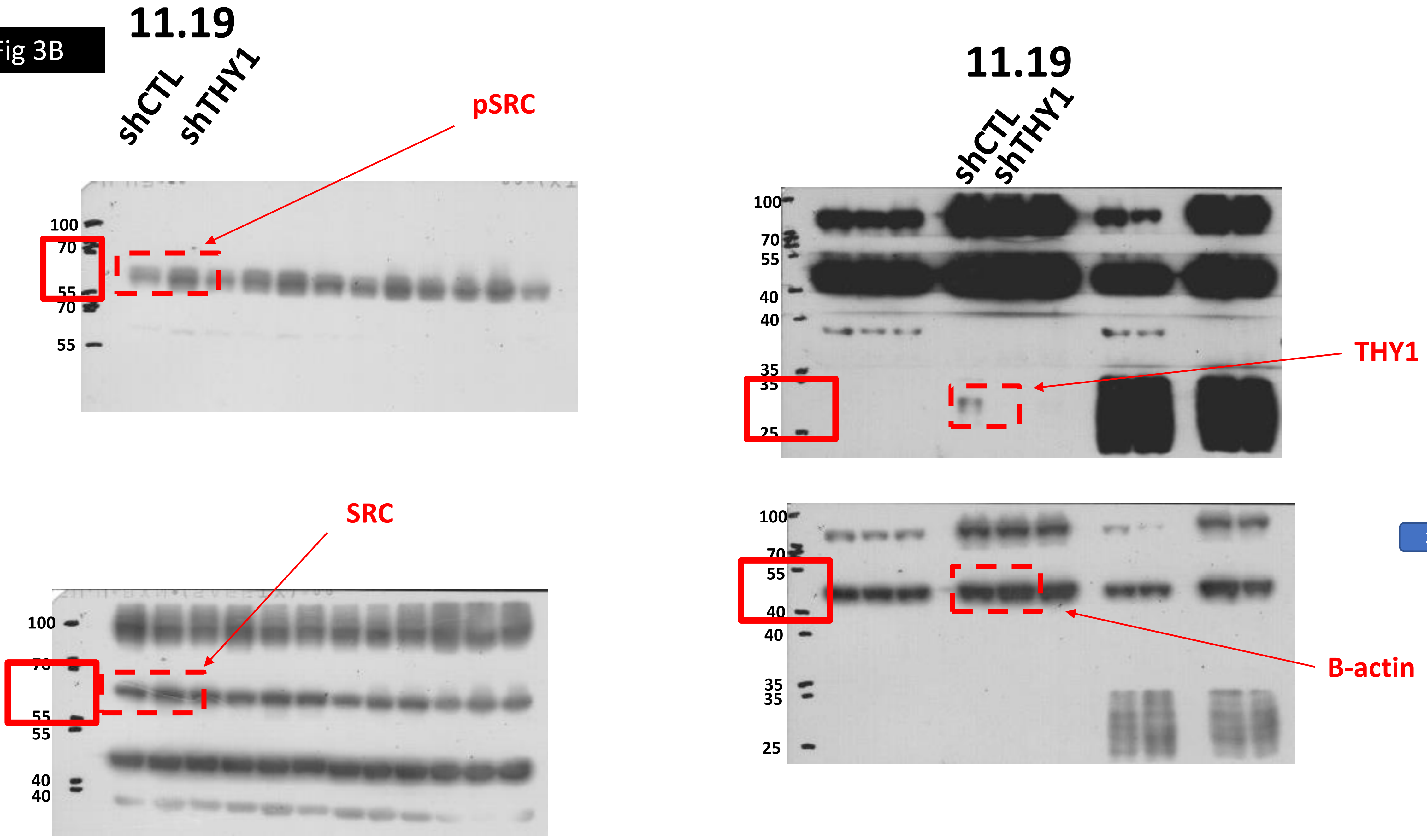

2017-09-22

Fig 3C

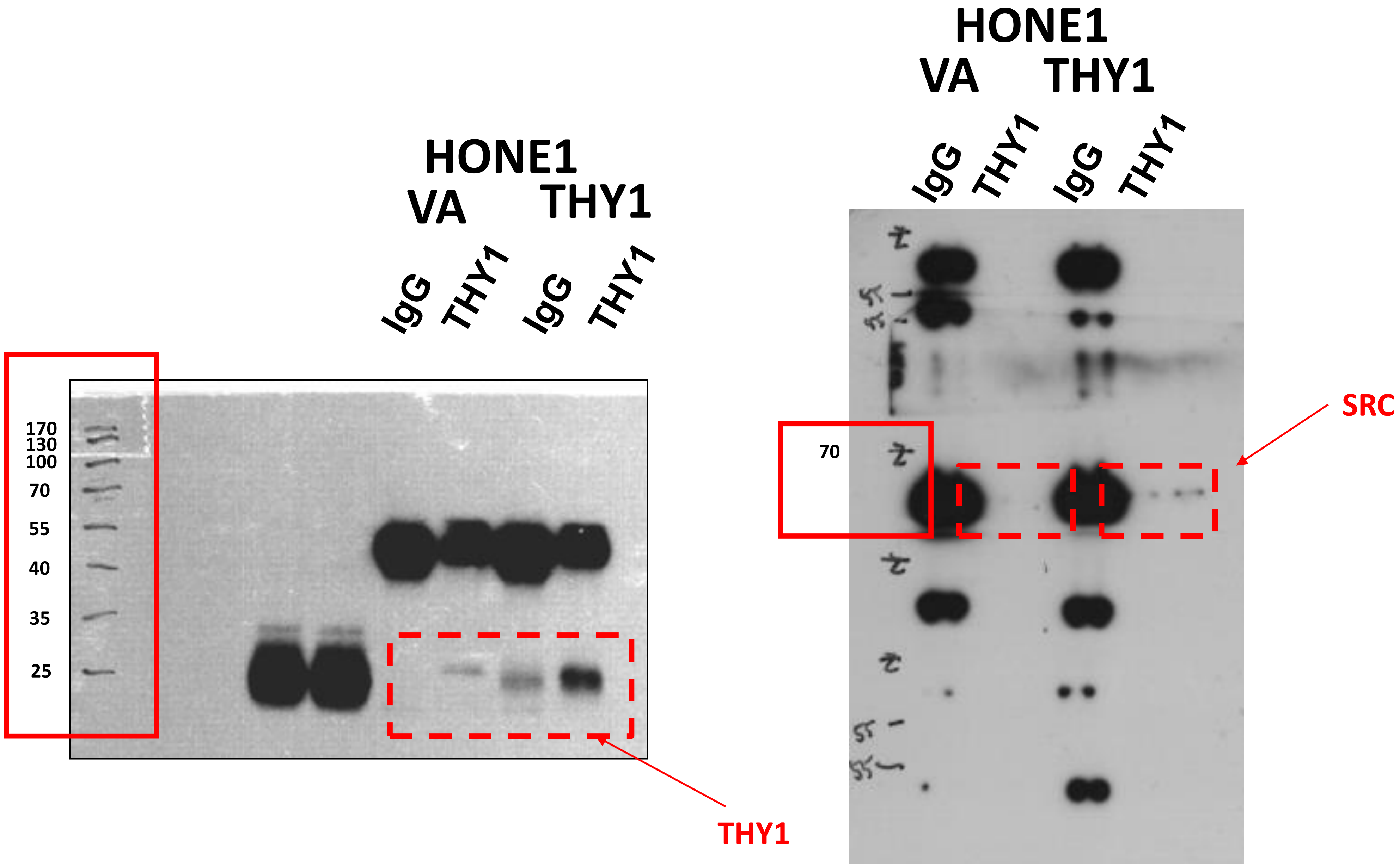
HONE1
VA
THY1
IgG
THY1
IgG
THY1
170
130
100
70
55
40
35
25
THY1
HONE1
VA
THY1
IgG
THY1
IgG
THY1
70
SRC

Fig 3D

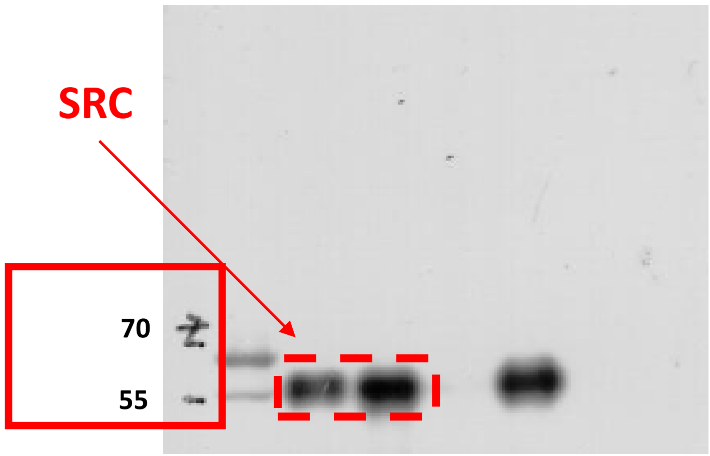

2018-10-03

Fig 3E

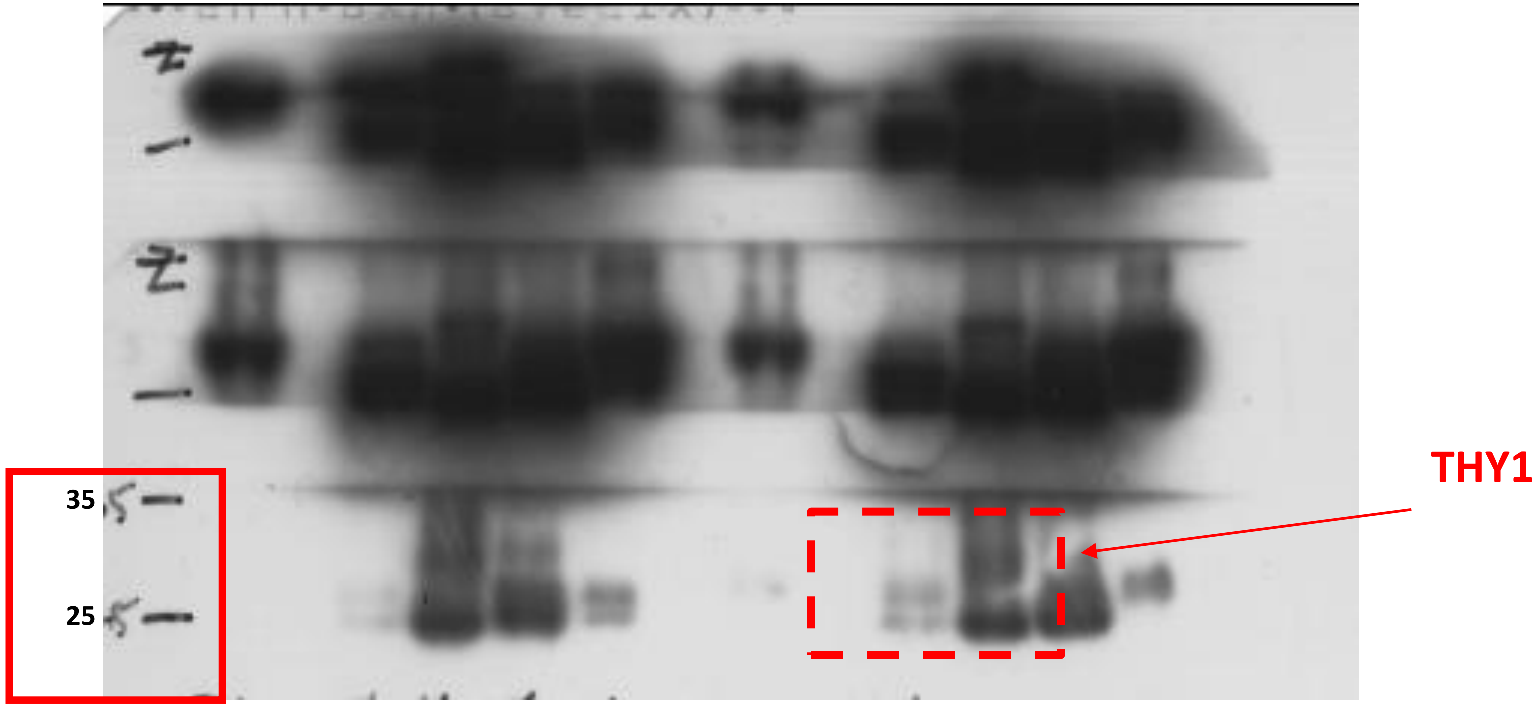

2017-10-11

Fig 3F

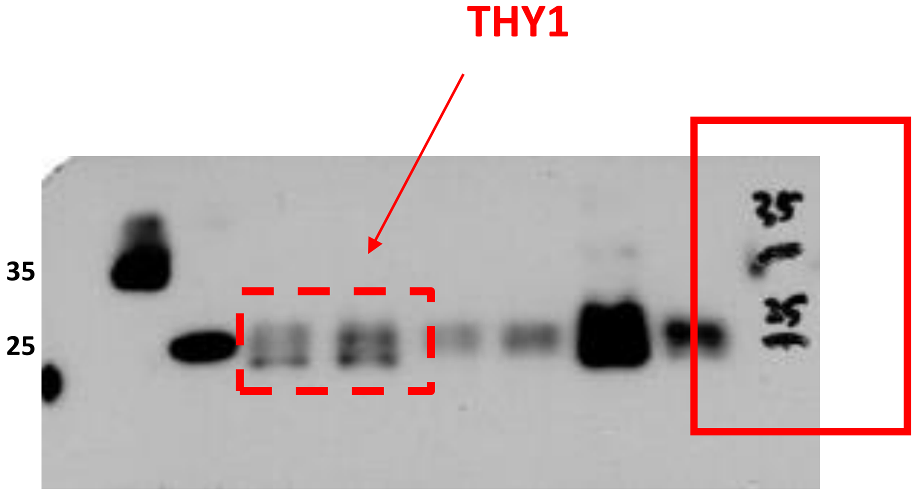

2017-10-03

Fig 4A

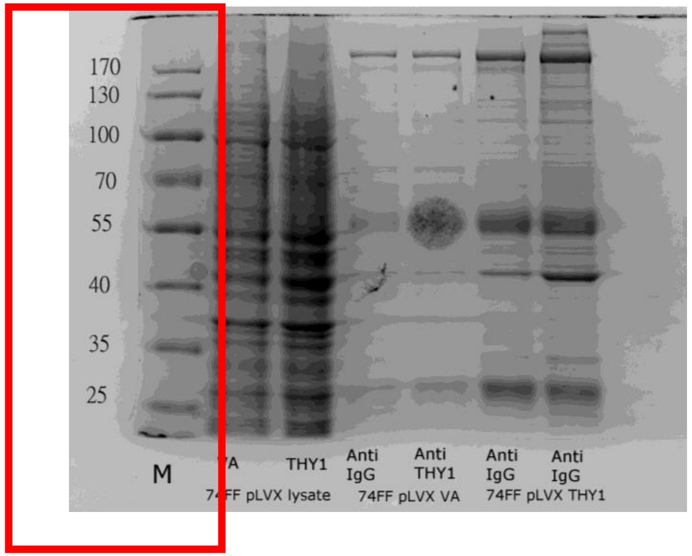
170
130
100
70
55
40
35
25
M
VA
THY1
Anti IgG
Anti THY1
Anti IgG
Anti IgG
74FF pLVX lysate
74FF pLVX VA
74FF pLVX THY1

Fig 4B

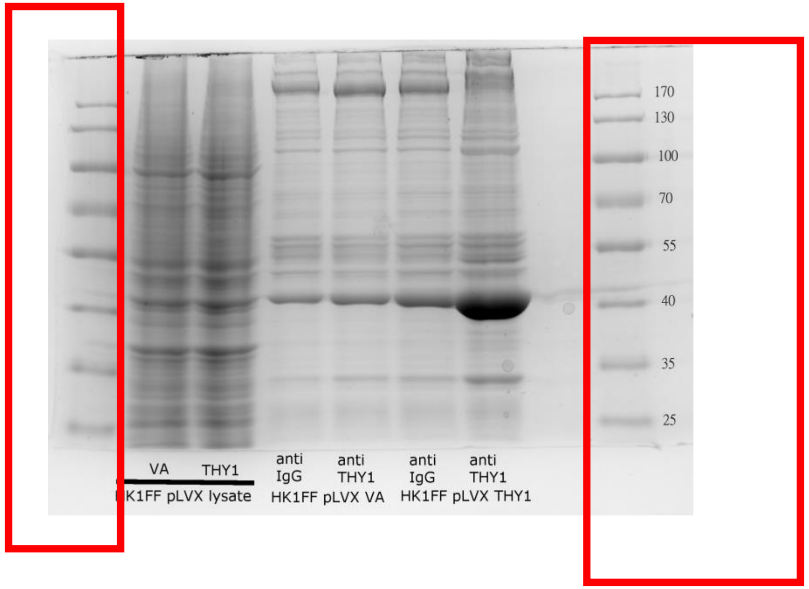
170
130
100
70
55
40
35
25
VA
THY1
HK1FF pLVX lysate
anti
IgG
anti
THY1
HK1FF pLVX VA
anti
IgG
anti
THY1
HK1FF pLVX THY1

Fig 4C

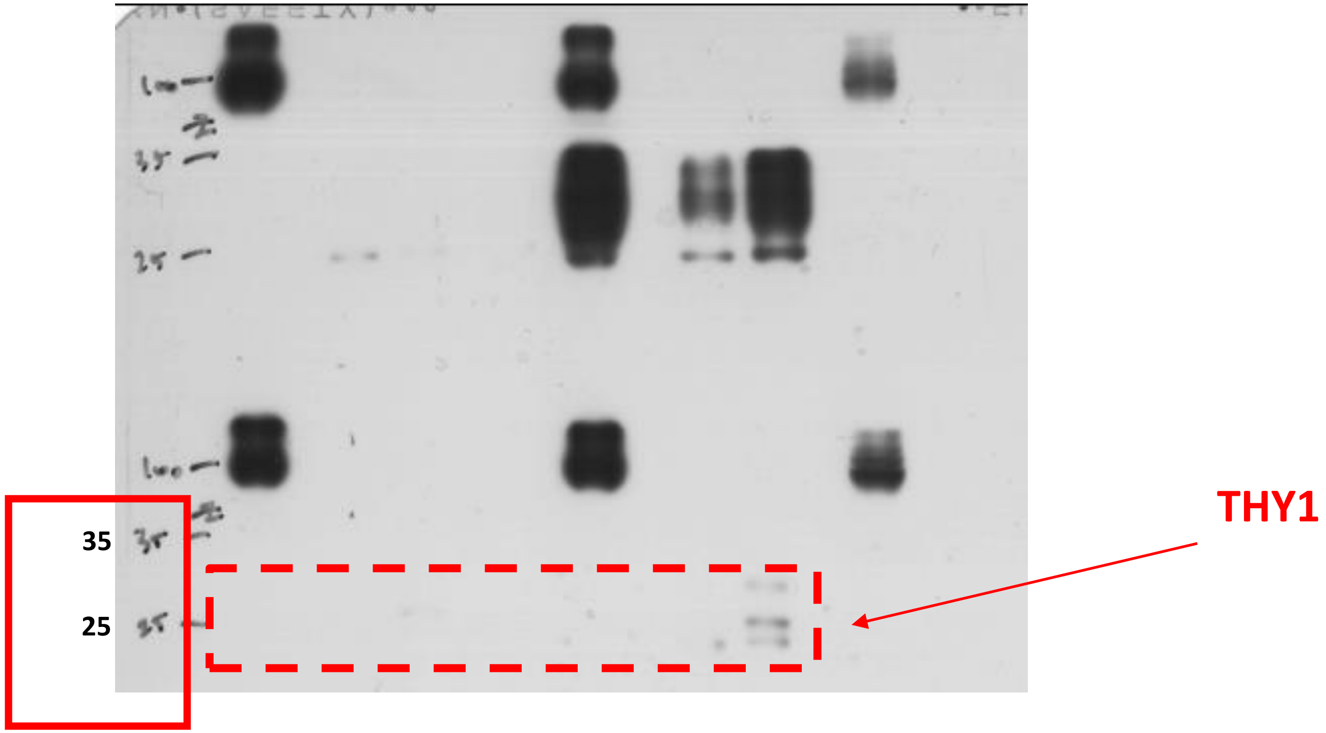

2017-11-07

Fig 4D

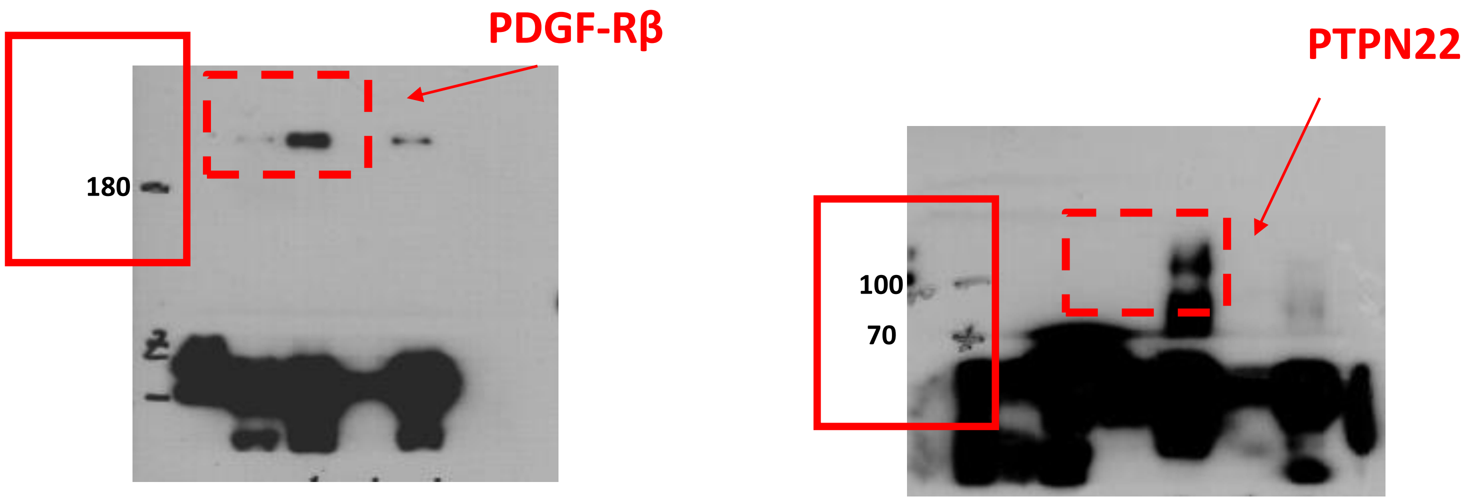

Fig 4F

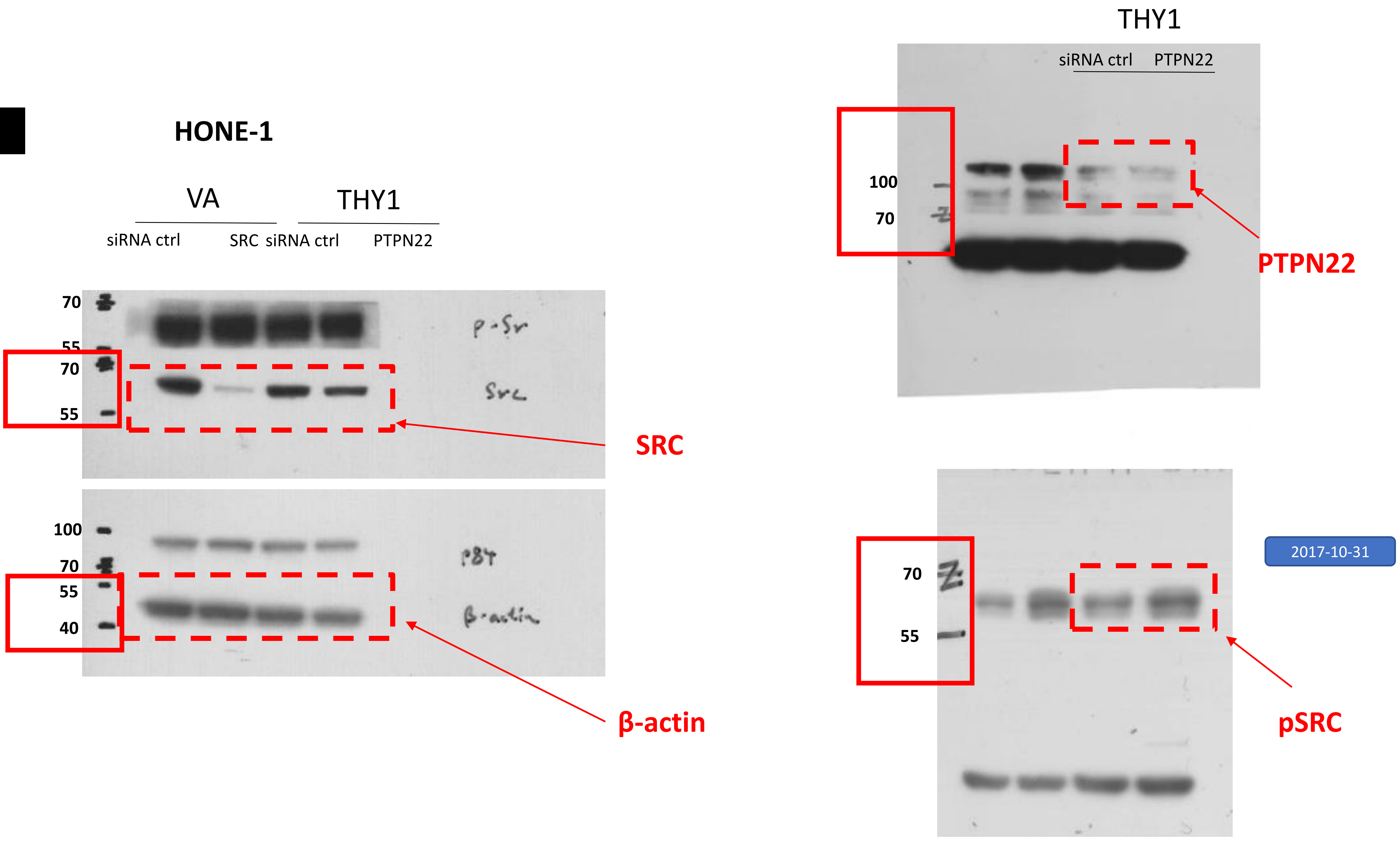
HONE-1
VA
THY1
siRNA ctrl
SRC
siRNA ctrl
PTPN22
70
55
70
55
SRC
100
70
55
40
β-actin
THY1
siRNA ctrl
PTPN22
100
70
PTPN22
70
55
pSRC
2017-10-31

Fig 4J

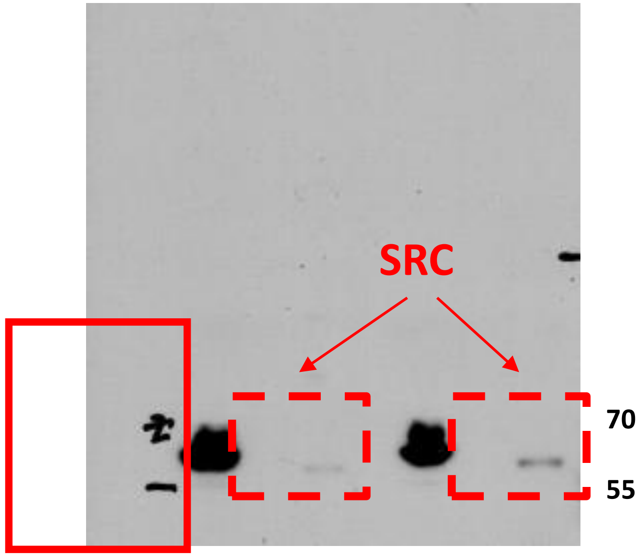

Fig 4K

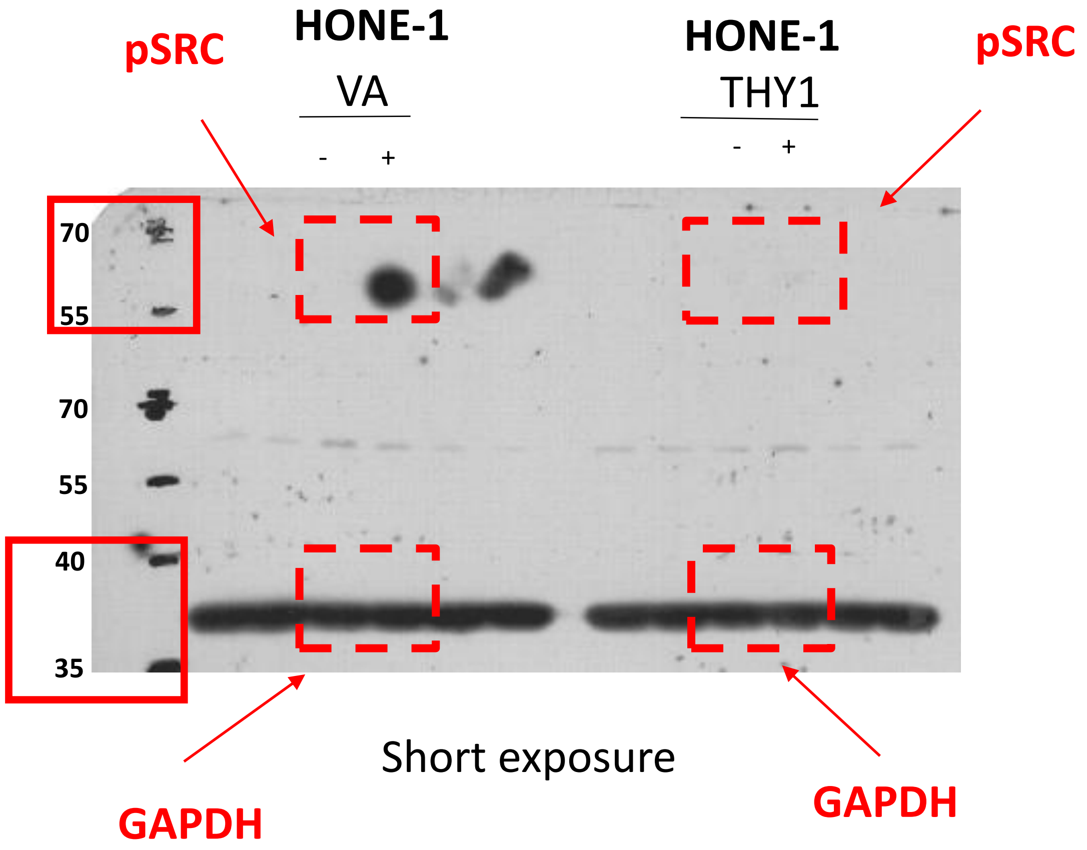

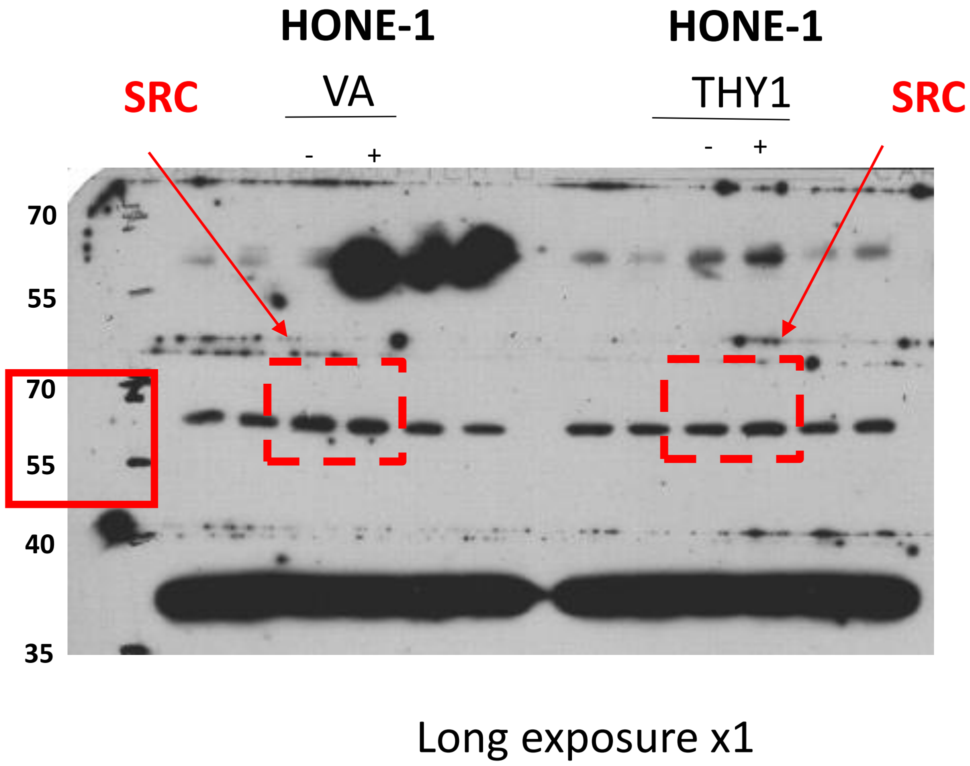

2018-10-30
